# Fragment Discovery by X‐Ray Crystallographic Screening Targeting the CTP Binding Site of *Pseudomonas Aeruginosa* IspD

**DOI:** 10.1002/anie.202414615

**Published:** 2025-01-02

**Authors:** Daan Willocx, Lucia D'Auria, Danica Walsh, Hugo Scherer, Alaa Alhayek, Mostafa M. Hamed, Franck Borel, Eleonora Diamanti, Anna K. H. Hirsch

**Affiliations:** ^1^ Helmholtz Institute for Pharmaceutical Research Saarland (HIPS) Helmholtz Centre for Infection Research (HZI) Campus E8.1 66123 Saarbrücken Germany; ^2^ Department of Pharmacy Saarland University Campus E8.1 66123 Saarbrücken Germany; ^3^ PharmaScienceHub Campus A 2.3 66123 Saarbrücken Germany; ^4^ Univ. Grenoble Alpes CEA CNRS, IBS F-38000 Grenoble France; ^5^ Helmholtz Institute for Pharmaceutical Research Saarland (HIPS) Helmholtz Centre for Infection Research (HZI) Campus E8.1 66123 Saarbrücken Germany

**Keywords:** Drug discovery, Fragment-based drug design, IspD, Medicinal chemistry, MEP pathway

## Abstract

With antimicrobial resistance (AMR) reaching alarming levels, new anti‐infectives with unprecedented mechanisms of action are urgently needed. The 2‐*C*‐methylerythritol‐D‐erythritol‐4‐phosphate (MEP) pathway represents an attractive source of drug targets due to its essential role in numerous pathogenic Gram‐negative bacteria and *Mycobacterium tuberculosis* (*Mt*), whilst being absent in human cells. Here, we solved the first crystal structure of *Pseudomonas aeruginosa* (*Pa*) IspD, the third enzyme in the MEP pathway and present the discovery of a fragment‐based compound class identified through crystallographic screening of *Pa*IspD. The initial fragment occupies the CTP binding cavity within the active site. Confirmation of fragment–protein interactions was achieved through ^1^H saturation–transfer difference nuclear magnetic resonance (^1^H‐STD NMR spectroscopy). Building upon these findings and insights from the co‐crystal structures, we identified two growth vectors for fragment growing. We synthesized derivatives addressing both growth vectors, which showed improved affinities for *Pa*IspD. Our new fragment class targets *Pa*IspD, displays promising affinity and favorable growth vectors for further optimization.

## Graphical abstract

During a crystallographic screening with IspD from *Pseudomonas aeruginosa*, we identified a fragment residing inside the active site. We confirmed its interactions with the protein by ^1^H saturation transfer difference NMR spectroscopy. Initial fragment growing yielded derivatives with improved affinity towards IspD from *Pseudomonas aeruginosa*.

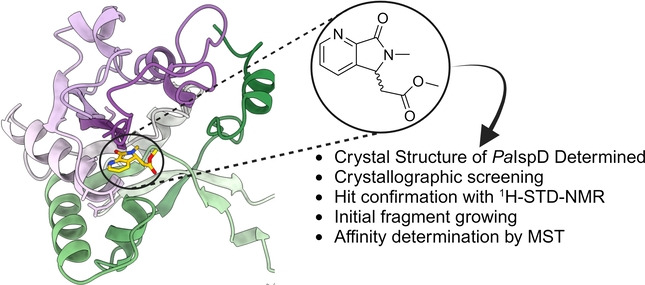

There have been few medical innovations as influential to present‐day life as the discovery of anti‐infectives. Not only do they allow cheap and straightforward treatment of infectious diseases, the confidence that infections could be treated enabled major leaps forward in other medical fields, including surgery and transplantations.[^1^, ^2^] However, we might find ourselves lacking potent anti‐infectives in the midst of the current antimicrobial resistance (AMR) crisis.^[3]^ Moreover, the clinical pipeline for new anti‐infectives has mostly dried up and most of the new antibiotics that do reach the market are modified agents of commercialized antibiotic classes, facing rapid resistance development.^[2]^ Furthermore, the vast majority of these new anti‐infectives are ineffective against infections caused by Gram‐negative bacteria, which make up a substantial fraction of hospital‐acquired infections.^[4]^ Therefore, there is an urgent need for new anti‐infectives with innovative modes of action, especially those able to target Gram‐negative bacteria. The 2‐*C*‐methylerythritol‐D‐erythritol‐4‐phosphate (MEP) pathway for the biosynthesis of the isoprenoid precursors isopentenyl diphosphate (IDP) and dimethylallyl diphosphate (DMADP) is a promising source of drug targets. The pathway is essential for a wide variety of pathogens, including *Mycobacterium tuberculosis* (*Mt*), *Pseudomonas aeruginosa* (*Pa*) and, *Klebsiella pneumoniae* (*Kp*), and is absent in human cells, minimizing the risk of off‐target side effects.^[5]^

In the present work, we set our focus on the third enzyme in the MEP pathway, namely IspD, catalyzing the formation of 4‐diphosphocytidyl‐2‐*C*‐methylerythritol (CDP‐ME) from MEP and CTP in the presence of Mg^2+^ (Scheme 1). To this day, the number of IspD inhibitors reported in literature is limited; moreover, the majority of these inhibitors focus on either plasmodial or botanically derived IspD.^[6]^ Consequently, there remains a considerable gap in our knowledge on inhibitors targeting bacterial homologues of IspD. Notably, one such unexplored variant is IspD originating from *Pa* (*Pa*IspD). In this study, we unveil, for the first time, the crystal structure of this enzyme (PDB accession code 9GBY, resolution: 1.50 Å), which enabled us to embark on a crystallographic fragment screening endeavor, leading to the identification of a co‐crystal structure in complex with a fragment hit. Subsequently, we confirmed interactions using ^1^H‐saturation‐transfer difference nuclear magnetic resonance (^1^H‐STD NMR spectroscopy) and initiated fragment growing.[^7^, ^8^] Lastly, we determined the affinity of all derivatives towards *Pa*IspD using microscale thermophoresis (MST) and fluorescence quenching.

**Scheme 1:**
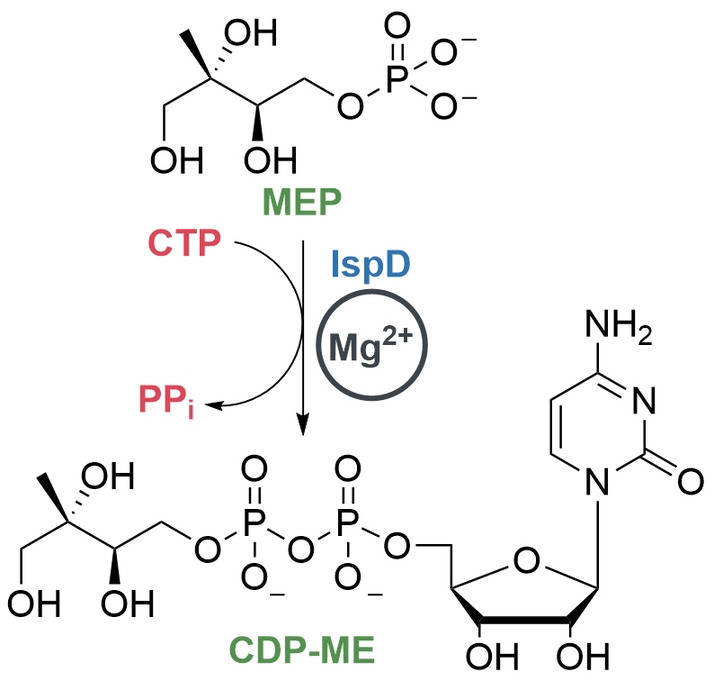
The reaction between 2‐*C*‐methylerythritol‐D‐erythritol‐4‐phosphate (MEP) and CTP catalyzed by IspD. Mg^2+^ is the presumed cofactor in this reaction.

Having established conditions for protein expression and purification and determined the Michaelis constant for both substrates (see Figure S1 Supporting Information), we began our investigation by obtaining an apo crystal structure of *Pa*IspD (PDB accession code 9GBY, without a cation), as no previous structures of *Pa*IspD were available in the RCSB Protein Data Bank (PDB). The structure was solved by molecular replacement using the previously published apo crystal structure of *Ec*IspD (PDB accession code: 1INJ) as a template.^[9]^ The solved *Pa*IspD structure belongs to the same space group (C2) and shows similar lattice parameters. The asymmetric units contain only one molecule and about 47 % solvent. Several IspD structures from various organisms are already present in the PDB and all these structures are highly conserved. *Pa*IspD exhibits the standard IspD fold, characterized by a single‐domain structure in an α/β conformation composed of a seven‐stranded β‐sheet with interconnected loops and α‐helices, which are connected to a subdomain known as the β‐arm of the protein (See Figure S7 in the Supporting Information, PDB accession code 9GBY). This β‐arm is primarily responsible for the dimerization of two IspD subunits by forming a hook‐like structure that connects the two monomers; the interface between the two constitutes the protein‘s active site, which remains solvent‐exposed. Next, we embarked on a screening campaign of a library containing 192 fragments obtained from Edelris (Lyon, France). We chose to pursue a fragment‐based drug discovery (FBDD) approach as fragments enable to focus on true multiparameter optimization to ensure drug‐like properties will be achieved.^[10]^ Furthermore, their small size allows them to bind in regions which are hard to target with small molecules, for example allosteric pockets. In total, we collected crystals for 51 fragments, from which we obtained a handful of co‐crystal structures. Among these, we identified three fragments, which were in complex with the protein (See Figure S8 in the Supporting Information). From these fragments, **1** immediately caught our attention as the fragment exhibited the most promising structure for further development (Figure 1, PDB accession code 9GC8). Upon closer inspection, we noticed that the fragment occupied a tight pocket that is occupied by the cytosine moiety of CTP in the co‐crystal structure of *Ec*IspD with CTP (PDB accession code:1I52).^[9]^ Since all residues involved in CTP binding are conserved between both homologues, we assumed that the cavity had the same function in *Pa*IspD. Within this cavity, the main interaction between **1** and the protein seems to be a hydrogen bond formed between the pyridyl nitrogen atom and the side chain hydroxyl group of Ser 89 (distance=2.6 Å, Figure 1). Interestingly, this hydroxyl group undergoes a similar interaction with one of the nitrogen atoms of the cytosine of CTP. The rest of the atoms of **1** do not seem to undergo any other obvious interactions, with the acetate tail of **1** being mostly solvent‐exposed (Figure 1). Based on that, we hypothesized that a similar binding mode could be obtained by truncating this part of the fragment. Nevertheless, to experimentally confirm our hypothesis, we synthesized **2** (Figure 2, Scheme S1 in the Supporting Information) and co‐crystallized it with *Pa*IspD under identical conditions. Importantly, as seen in Figure 3, a matching binding mode was observed, thus confirming our hypothesis (PDB accession code 9GCA). Further, the smaller size of fragment **2** compared to **1**, allowed us to improve the ligand efficiency parameter, a key aspect in fragment‐based drug discovery.

**Figure 1:**
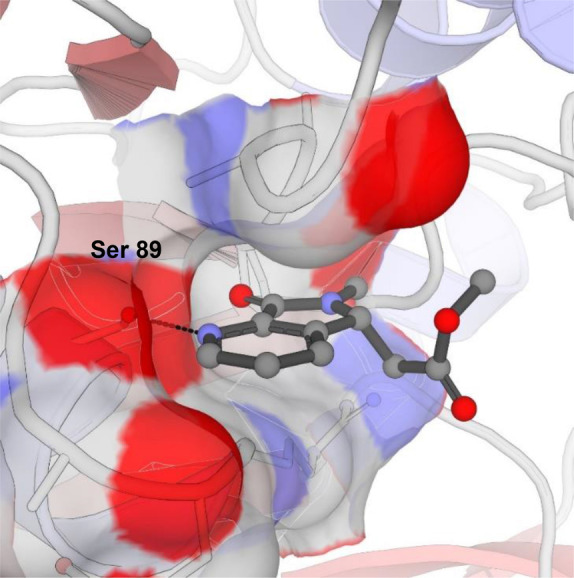
Co‐crystal structure of **1** within the active site of *Pa*IspD displaying its interaction with Ser‐89 (PDB accession code 9GC8).

**Figure 2:**
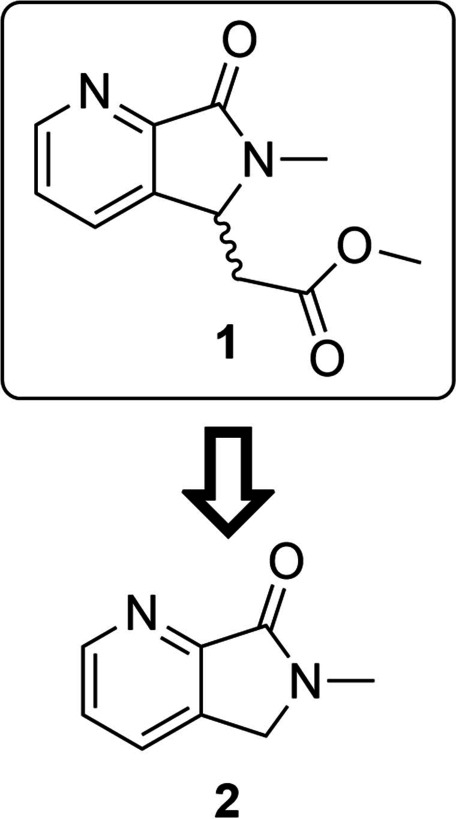
Chemical structure of **1** and its simplified derivative **2**.

**Figure 3:**
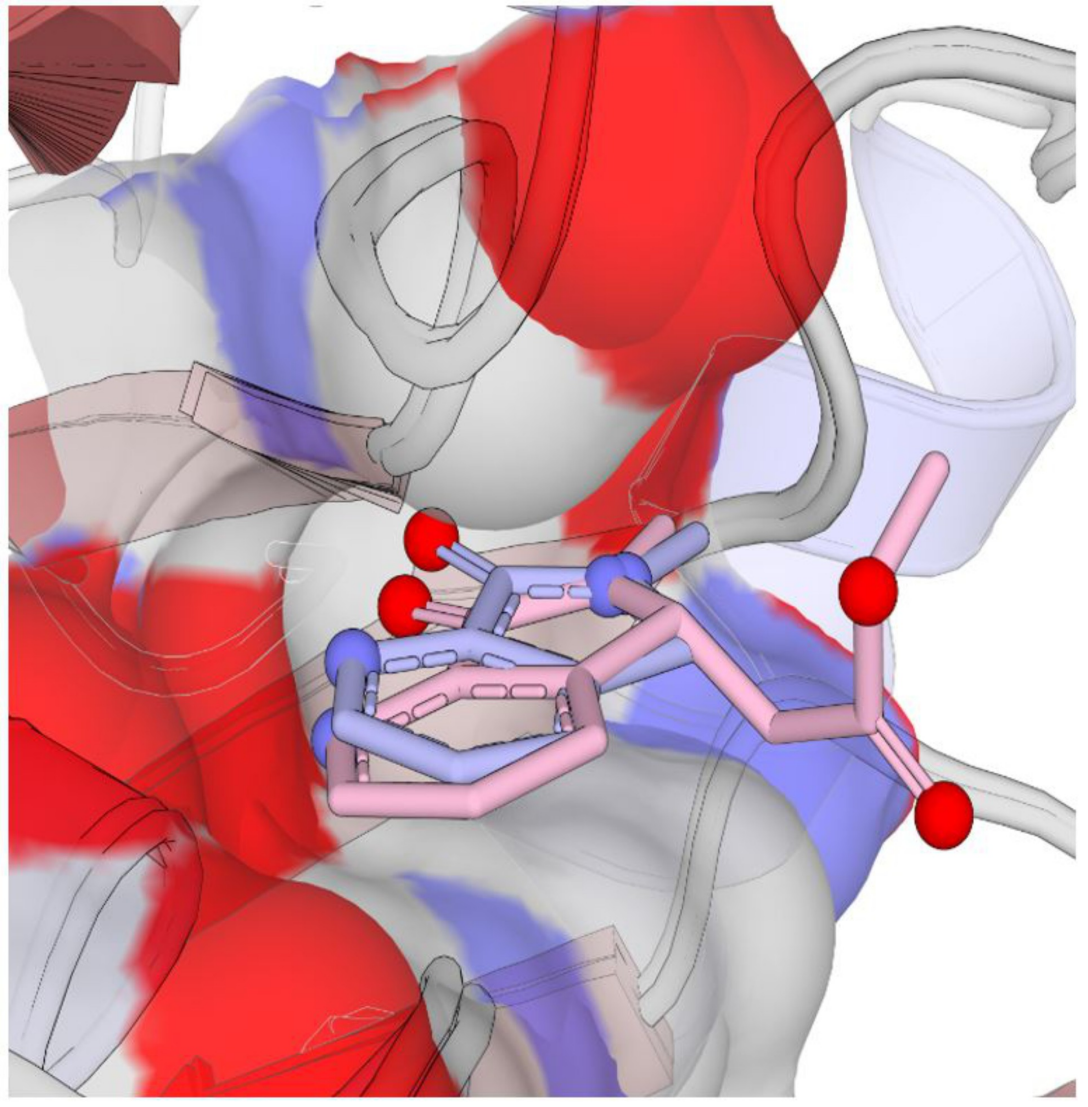
Comparison of the binding pose of **1** (pink) and **2** (light blue) in the active site of *Pa*IspD (PDB accession codes 9GC8 and 9GCA).

To further validate the occurrence of interactions taking place between the fragment and *Pa*IspD, we referred to ^1^H‐STD NMR spectroscopy, a commonly used technique for this purpose.[^10^, ^11^] We conducted this experiment in a HEPES buffer in D_2_O with a 100‐fold excess of **2** compared to *Pa*IspD. In the corresponding difference spectrum, we identified all proton signals previously observed in the ^1^H NMR spectrum of **2** (see Figure S2 and S3 in the Supporting Information). This observation suggests that **2** came into close proximity of the protein, thereby affirming the potential interaction between them. We further noted the magnitude of amplification of the signals as the efficiency of saturation transfer scales with distance, enabling us to gain an idea about the proximity of each proton to the protein surface.^[12]^ Among all protons, those in position **iii** (Figure 4) clearly received a surplus in saturation transfer relative to the other ones, whereas the protons positioned on the pyridyl ring (**i**, Figure 4) and the protons in position **ii** (Figure 4) received both less amplification with the latter receiving the least amount (Figure 4). These observations correlate with the pose observed inside the co‐crystal structure, although, based on the crystal structure, we expected to see a difference in amplification between the protons residing on the pyridyl ring, with the proton next to the pyridyl nitrogen atom receiving more saturation than the other protons present on the ring. We might explain the observed difference with the static environment of co‐crystal structures in comparison with the dynamic environment in solution during a ^1^H‐STD NMR experiment.

**Figure 4:**
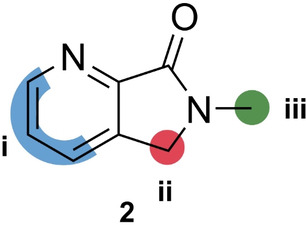
Indication of the areas that received saturation transfer during the ^1^H saturation transfer difference (STD) NMR experiment. Blue: region **i**; Red: region **ii**; Green: region **iii**.

To confirm that **2** binds to the CTP binding site, we resorted to competition STD NMR experiments performed between **2** and CTP in the presence of *Pa*IspD.^[13]^ At first, we kept the concentration of both **2** and CTP equal at 1 mM. This resulted in a difference spectra where signature peaks of both competitors could be observed (Figure S5 in the Supporting Information). When we then increased the concentration of CTP tenfold in comparison to **2**, the STD signals of **2** disappeared, thus indicating that both ligands compete for the same binding site (Figure S6 in the Supporting Information).

Having validated the binding mode of our fragment hit (**2**), we embarked on a rational fragment growing campaign. Analysis of the co‐crystal structure and the ^1^H‐STD NMR spectrum suggested two potential growth vectors. Based on the co‐crystal structure, modifications in position **iii** (Figure 4) seemed most straightforward, as this would extend the fragment inside the active site towards the location where the ribose of CTP is bound. However, the protons in this position received significant amplification in the ^1^H‐STD NMR spectrum, implying that the methyl is in close proximity to the protein surface. On the other hand, based on the ^1^H‐STD NMR spectrum, modifications in position **ii** (Figure 4) seem most logical as the corresponding protons only received limited amplification. Although we previously stated that modifications in this position point towards the solvent in the co‐crystal structure, we anticipated that with a different linker some interactions could still occur as the protein surface is in the vicinity. We preferred to initially design and synthesize a focused subset of derivatives containing modifications in both positions (Tables 1 and 2) and assess them using MST and fluorescence quenching. To see if our modifications had a positive influence on the affinity for *Pa*IspD, we first assessed **2** by both techniques as reference. Unfortunately, a reliable *K*_d_ value could not be determined with MST as aggregation occurred at high concentrations before a complete dose‐response curve could be established. Despite this set‐back, we proceeded with fragment growing, hoping to improve affinity and solubility. To avoid potential clashes with the protein surface when growing in position **iii** (Figure 4), we attached most derivatives *via* an alkyl linker to allow some flexibility (Table 1). Modifications on this side of the molecule seem to be tolerated and led to an improvement of the affinity towards the protein in comparison with **2** (**2**, *K*_d_ by Δ fluo=977±59 μM; **4**, *K_d_* by Δ fluo=391±35 μM). At this point in time, it is unsure if a linker is critical for growth at this vector with **3** displaying comparable activity as **4** during our assessment using MST (**3**, *K*_d_ MST=186±7 μM, **4**, *K*_d_ MST=133±12 μM), while when using fluorescence quenching, a large difference is observed (**3**, *K*_d_ Δ fluo=879±64 μM; **4**, *K_d_* Δ fluo=391±35 μM). For modifications in position **ii** (Figure 4), we opted to use methylamine as a linker between the core of the fragment and tail instead of a methyl (see Scheme S2 in the Supporting Information). This linker not only has the ability to form hydrogen bonds with the protein itself, but is also more chemically accessible enabling straightforward modifications via nucleophilic addition or substitution reactions. At first, we synthesized the methylcarbamate **7** starting from the methylamine in an attempt to closely mimic **1** (Table 2 and Scheme S3 in the Supporting Information). The attachment of the tail containing the methylcarbamate moiety improved the affinity of the initial fragment hit **2** (**2**, *K*_d_ Δ fluo=977±59 vs **7**, *K_d_* MST=454±27 μM). Encouraged by these results, we synthesized compounds **8**‐**12** (see Table 2, Scheme S3 and Scheme S4 in the Supporting Information). Although most of these derivatives showed an improvement in affinity compared to **2**, compounds **10** (*K_d_* MST=209±4 μM) and **12** (*K_d_* MST=239±6 μM) were the best in the series (Table 2). The fact that the affinity of the fragment for *Pa*IspD could be improved by modifications at either growth vector is highly encouraging for further optimization of this fragment class. To gain insights on the orientation of these new derivatives in the active site, we attempted to co‐crystalize them with *Pa*IspD. We were only partially successful with this approach, obtaining co‐crystal structures with compounds **4**, **5**, **8**, **10** and, **12**. Unfortunately, within the electron density maps of these structures, we could only observe partial density for the compounds, despite them residing in the same pocket as **1** and **2** (Figures S**9**, **10** and **11** in the Supporting Information).

**Table 1:** Dissociation constants (*K_d_*) towards *Pa*IspD for **2**–**6**, bearing modifications at position **iii**. Ligand efficiencies for **2**–**6**.

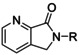				
#	Structure, R=	MST *K*_d_ [μM]^[a]^	Δ fluo *K*_d_ [μM]^[a]^	Ligand efficiency ^[b]^
2	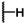	939±39^[c]^	977±59	−1.35
3	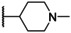	186±7	879±64	−0.77
4	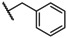	133±12	391±35	−0.97
5	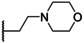	491±2	ND^[d]^	−0.73
6	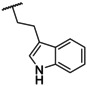	208±7	332±22	−0.79

[a] Assays were performed in replicate as independent experiments (*n*=2). values are shown as mean±SD. [b] Calculated via the following formula: *Ligand efficiency=1.37* p*K*_d_ */ HA*, in which *K*_d_=MST *K*_d_ and HA=non‐hydrogen (heavy) atom. [c] Insufficient compound stock. *K*_d_=dissociation constants. Δ fluo=Fluorescence quenching. ND=not determined.

**Table 2:** Dissociation constants (*K_d_*) towards *Pseudomonas aeruginosa* (*Pa*) IspD for **7**–**12**, bearing modifications in position **ii**. Ligand efficiencies for **7**–**12**.

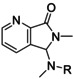									
#	Structure, R=	MST *K*_d_ [μM]^[a]^	Δ fluo *K*_d_ [μM]^[a]^	Ligand efficiency^[b]^	#	Structure, R=	MST *K*_d_ [μM]^[a]^	Δ fluo *K*_d_ [μM]^[a]^	Ligand efficiency^[b]^
7	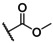	454±27	ND^[c]^	−0.72	10	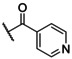	209±4	310±28	−0.63
8	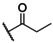	ND	248±15	ND	11	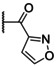	502±56	301±16	−0.61
9	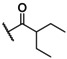	536±47	ND^[c]^	−0.93	12	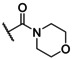	239±6	371±42	−0.54

[a] Assays were performed in replicate as independent experiments (*n*=2). values are shown as mean±SD. [b] Calculated via the following formula: *Ligand efficiency=1.37* p*K*_d_ */ HA*, in which *K*_d_=MST *K*_d_ and HA=non‐hydrogen (heavy) atom. [c] slight aggregation occurred in one of the measurements at high concentration before a complete dose‐response curve could be established. [d] Insufficient compound stock. *K*_d_=dissociation constant. Δ fluo=Fluorescence quenching. ND=Not Determined.

In summary, we report for the first time the crystal structure of *Pa*IspD (PDB accession code 9GBY, resolution: 1.50 Å) along with the discovery of a fragment‐based compound class uncovered during a crystallographic fragment screening using *Pa*IspD (PDB accession code 9GC8, resolution: 1.95 Å). Noteworthy, the co‐crystal structures (PDB: 9GC8 and 9GCA) between *Pa*IspD and fragments **1** and **2** unveil a binding mode for this chemical class that has never been reported until now for this target enzyme. So far, only Witschel *et al*. reported a co‐crystal structure of a plant homologue (*Arabidopsis thaliana* IspD) with a fragment bound to an allosteric binding site.^[14]^ Here, we managed to handle a human pathogen organism (*P. aeruginosa*) and to target directly the active site of the protein with a fragment that possess excellent drug‐like properties. We confirmed interactions between the protein and the fragment by employing ^1^H‐STD NMR spectroscopy. Consolidation of the competitive behavior of the fragment towards CTP was obtained via competitive STD NMR experiments. Combining these results with the insights obtained from the co‐crystal structure led to the identification of two potential growth vectors. We synthesized derivatives containing modifications at either growth vector and assessed them using MST and fluorescence quenching. Modifications on both sides led to an improvement in affinity towards *Pa*IspD, validating both growth vectors. We believe this fragment binding to *Pa*IspD could be a valuable addition to the search for new compounds that target Gram‐negative pathogens with a novel mechanism of action. Furthermore, due to the high degree in conservation of the active site among IspD originating from different Gram‐negative pathogens, the fragment could play a role in addressing those pathogens as well.^[8]^

## Conflict of Interests

The authors declare no conflict of interest.

## Supporting information

As a service to our authors and readers, this journal provides supporting information supplied by the authors. Such materials are peer reviewed and may be re‐organized for online delivery, but are not copy‐edited or typeset. Technical support issues arising from supporting information (other than missing files) should be addressed to the authors.

Supporting Information

Supporting Information

## Data Availability

The data that support the findings of this study are available in the supplementary material of this article.
