# Supplementary material for: Fragment Discovery by X‐Ray Crystallographic Screening Targeting the CTP Binding Site of Pseudomonas Aeruginosa IspD
Source: Angew Chem Int Ed Engl. 2025 Jan 2;64(6):e202414615. doi: 10.1002/anie.202414615 (PMC11796317; doi:10.1002/anie.202414615)
Supplement: Supplementary file 2 — Supporting Information [file ANIE-64-e202414615-s002.pdf]

Angewandte Chemie
Eine Zeitschrift der Gesellschaft Deutscher Chemiker

Supporting Information

Fragment Discovery by X-Ray Crystallographic Screening Targeting the CTP Binding Site of *Pseudomonas Aeruginosa* IspD

D. Willocx, L. D'Auria, D. Walsh, H. Scherer, A. Alhayek, M. M. Hamed, F. Borel, E. Diamanti*, A. K. H. Hirsch**

Supporting Information

Fragment Discovery by X-ray Crystallographic Screening Targeting the CTP Binding Site of *Pseudomonas aeruginosa* IspD

Daan Willocx,[a] Lucia D'Auria,[b] Danica Walsh,[c] Hugo Scherer,[a] Alaa Alhayek,[a] Mostafa M. Hamed,[c] Franck Borel,* [b] Eleonora Diamanti,*[c] Anna K. H. Hirsch*[a]

[a] D. Willocx, H. Scherer, Dr A. Alhayek, Prof. A. K. H. Hirsch
Helmholtz Institute for Pharmaceutical Research Saarland (HIPS), Helmholtz Centre for Infection Research (HZI)
Campus E8.1,66123 Saarbrücken (Germany)
and
Department of Pharmacy, Saarland University
Campus E8.1,66123 Saarbrücken (Germany)
E-mail: Anna.Hirsch@helmholtz-hips.de

[b] Dr. L. D'Auria, Dr. F. Borel
Univ. Grenoble Alpes, CEA, CNRS, IBS, F-38000 Grenoble (France)
E-mail: franck.borel@ibs.fr

[c] Dr. D. Walsh, Dr. M. M. Hamed, Dr. E. Diamanti,
Helmholtz Institute for Pharmaceutical Research Saarland (HIPS), Helmholtz Centre for Infection Research (HZI)
Campus E8.1,66123 Saarbrücken (Germany)
E-mail: eleonora.diamanti2@unibo.it

Table of Contents

Crystalization conditions S1
Determination of enzyme kinetics S2
Saturation-Transfer Difference (STD)-NMR. S3
Competition STD-NMR. S5
MST Procedure (*Pa*IspD) S7
Fluorescence quenching (*Pa*IspD) S8
Chemistry S9
Figures, Schemes and Tables S12
Electron density maps of compounds 4, 5, 8, 10 and 12 .... S15
^{1}H NMR, ^{13}C NMR and LC-MS Spectra of Final Compounds S16
Heated NMR Spectra of 12 .... S40
References S42

Crystalization conditions

***Pa*IspD expression and purification.** The *Pseudomonas Aeruginosa* IspD (*Pa*IspD) synthetic gene was cloned into pET28 expression plasmid. *Pa*IspD, fused to an *N*-termimal His6 -tag, was expressed in *Escherichia coli* BL21 (DE3) strain. Cell cultures were grown in LB medium at 37 °C until an optical density (A600) of 0.7; then protein expression was induced by adding isopropyl β-D-thiogalactopyranoside to a final concentration of 1 mM. Expression was carried out for four hour at 37 °C. Cells were pelleted by centrifugation, resuspended in 50 mM NaH_2PO_4 pH 8.0, 300 mM NaCl, 1 % tween-20, 20 mM imidazole, 10mM β-mercaptoethanol,10% glycerol and Complete EDTA-free antiprotease (Roche Diagnostics, Meylan, France) (1 tablet Complete in 50 mL buffer), and lysed by sonication. Cellular debris were removed by centrifugation (60 min, 15 000 g). After centrifugation, the supernatant was applied on a Nickel beads (Ni-NTA, Qiagen) previously equilibrated in the lysis buffer. The column was washed with 50 mM NaH_2PO_4 pH 8.0, 300 mM NaCl, 40 mM imidazole, 10% glycerol, 10 mM β-mercaptoethanol. *Pa*IspD was eluted with 250 mM imidazole. Incubation with thrombin during overnight dialysis at 4 °C against lysis buffer minus tween-20 removed the amino terminal His-tag. Dialysed protein was reloaded on a Ni-NTA column. The flow-through was depleted of thrombin using a benzamidine-sepharose column. The protein was further purified by gel filtration on a HiLoad 16/60 Superdex-200 prep grade column equilibrated and eluted with 50 mM Hepes pH 7.5, 100 mM NaCl and 2 mM DTT. Fractions containing *Pa*IspD were pooled, concentrated to 15 mg/mL and stored at -80 °C.

(Co)-crystallization of the fragments with *Pa*IspD. The co-crystallization of *Pa*IspD with the chemical compounds of interest was carried out using "dry" coated crystallization plates. Prior to crystallization, the DropGuard supports of the 15-well EasyXtal® tools (Nextal) were filled with 1 or 2 µL of the chemical compounds to be co-crystallized (20-50 mM in DMSO) and then left in the open air until the DMSO had completely evaporated. The crystallization drops are then prepared by mixing 1 µL of the reservoir with 1 µL of the protein solution on the dried compound. IspD complex were crystallized by the hanging drop vapor diffusion method at 20 °C using either 26% PEG 500 MME or 26 % PEG 400, 0.1 M MES pH 6.5 and a protein concentration of 15 mg mL^{-1}. Native data sets were collected on BL13 XALOC at Alba synchrotron (Barcelona, Spain) and ID30A-3 beamline at ESRF (Grenoble, France). The diffraction data were processed using XDS. Crystals belong to the C2 space group and contain one molecule per asymmetric unit. The structures were solved by molecular replacement using Phaser and *E. coli* IspD structure (Protein Data Bank entry 1INJ)[15] as a search model. Model building was made using Coot and refinements were carried out with Phenix refine. Data and refinement statistics are summarized in table S1. The coordinates and the structure are deposited in the Protein Data Bank in Europe (http://www.pdbe.org) with the accession codes 9GBY (apo), 9GC8 (with **1**) and 9GCA (with **2**). [16]

Determination of enzyme kinetics

Michaelis constant (K_m) of both substrates was calculated and shown in Table S1 and Figure S1.

Table S1. Michaelis constants for MEP and CTP of *Pa*IspD.

Substrate	K_m (mM)	V_{max} (AU/min^{-1})
MEP	0.21 ± 0.1	0.30 ± 0.0
CTP	0.20 ± 0.1	0.30 ± 0.01

Determination of enzyme kinetics. The assay was conducted as published before.[17] To determine the K_m values for CTP and MEP with *Pa*IspD, substrate concentrations (0.016–2 mM) were prepared in a 96-well plate. Each well contained 100 µL of reaction mix with **Buffer A** (100 mM Tris-HCl pH 7.6, 1 mM DTT, 1 mM $MgCl_2$) and 50 nM *Pa*IspD. Serial dilutions of CTP or MEP were performed, and reactions were initiated by adding **Buffer B** (100 mM Tris-HCl pH 7.6, 1 mM DTT, 1 mM MEP or CTP). Plates were incubated at 37 °C for 40 minutes, denatured at 95 °C for 5 minutes, and centrifuged at 4000 rpm at 4 °C. Supernatants (10 µL) were mixed with an 190 µL ice-cold 3:1:1 ACN, isopropanol, water mixture containing 100 nM 4-methyl-1-oxo-1-(*p*-tolylamino)pentane-2-sulfonic acid, adenylyl-imidodiphosphate and adenosine-5′-[(α,β)-methyleno]triphosphate as internal standard. After centrifugation at 4000 rpm at 4 °C for 5 min, a 50 µL aliquot was transferred to an LC-MS plate for analysis. Peak areas were used to calculate Michaelis–Menten kinetics using GraphPad Prism v9. Measurements were done in duplicates and repeated in two independent experiments.

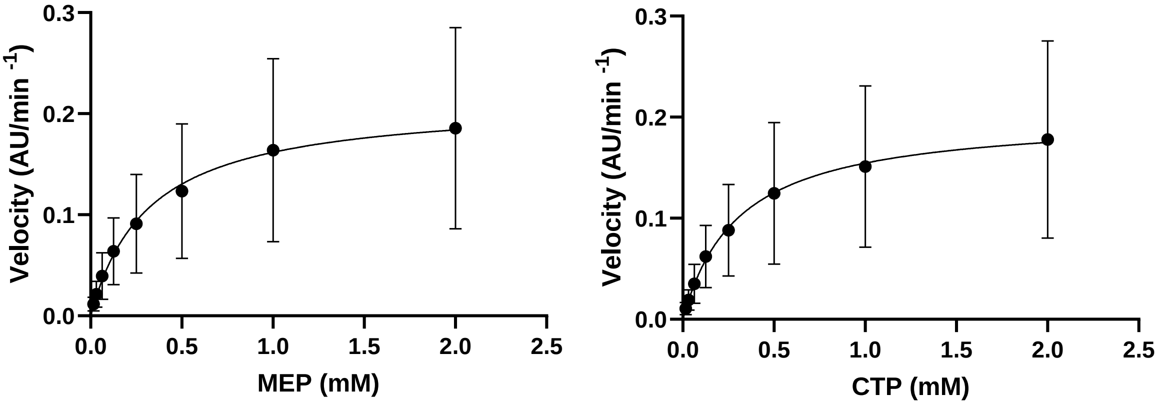

Figure S1: Kinetic parameters of *Pa*IspD substrates (MEP, left and CTP, right). Each data point reflects the mean ± SD obtained from two-independent experiments.

Saturation-Transfer Difference (STD)-NMR.

The ^{1}H-STD-NMR experiment was recorded at 25 ºC with 512 scans on a Bruker Avance Neo 500 MHz spectrometer with prodigy cryoprobe system. The on-resonance irradiation was set at -4 ppm, while the off-resonance was set to -40 ppm. Samples contained a concentration of 1 mM of **2** and 10 µM of *Pa*IspD resulting in a 100:1 ratio regarding the enzyme. Spectra were processed using Topspin 4.2.0, Bruker's NMR Data Analysis software.

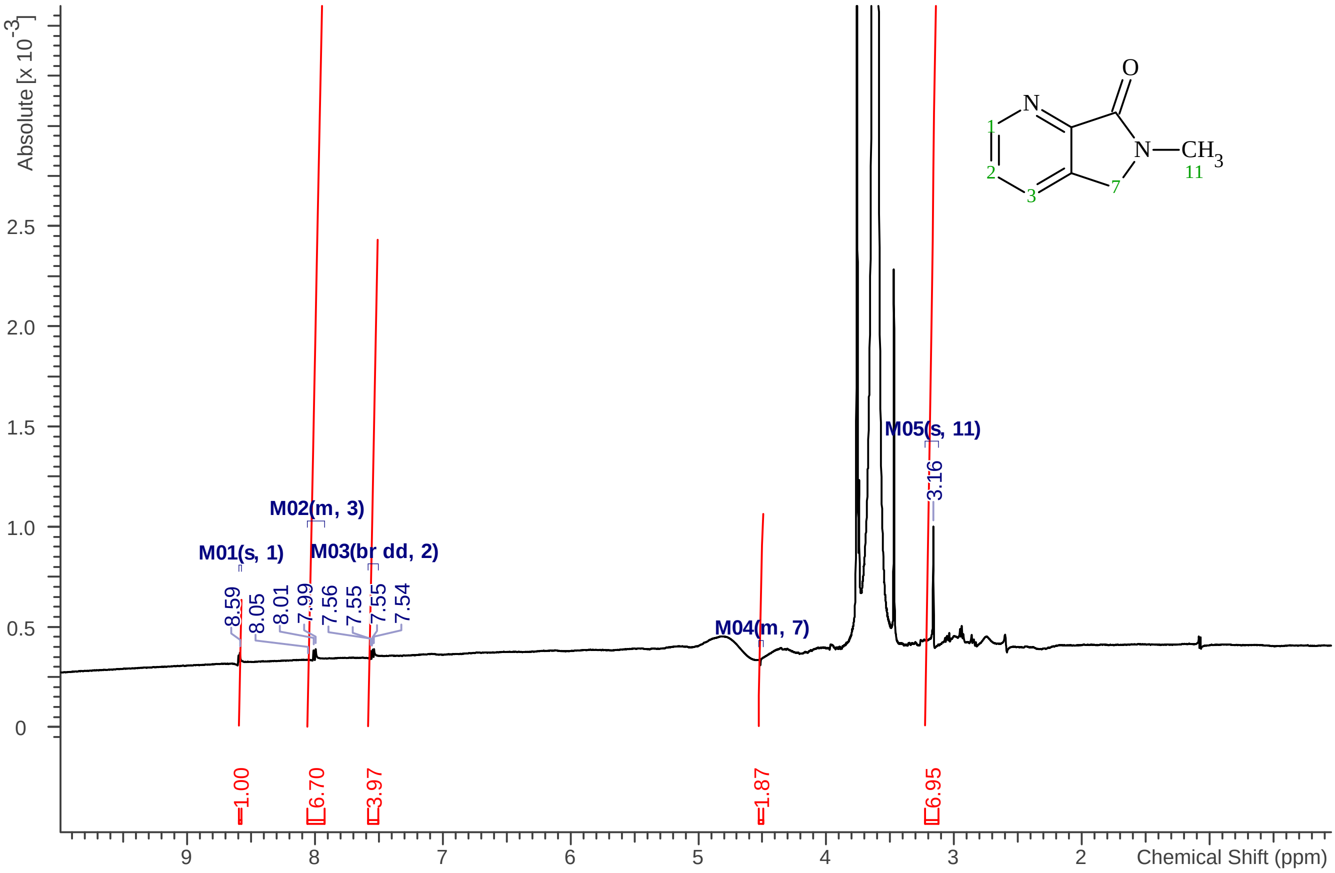

Figure S2. ^{1}H-STD-NMR spectrum of **2** with *Pa*-IspD.

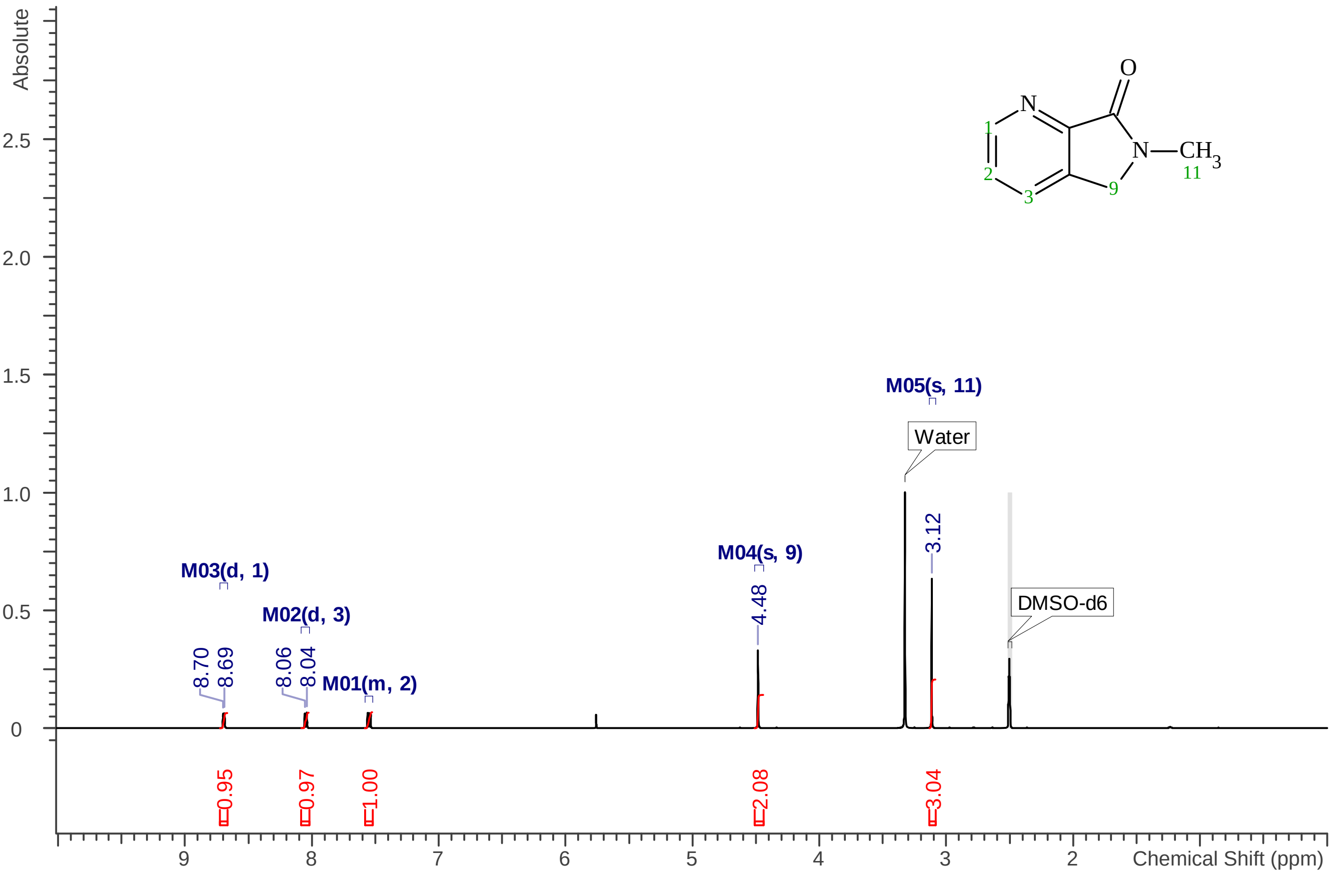

Figure S3. ^{1}H-NMR spectrum of **2**.

Competition STD-NMR.

The ^{1}H-STD-NMR experiment was recorded at 25 ºC with 512 scans on a Bruker Avance Neo 500 MHz spectrometer with prodigy cryoprobe system. The on-resonance irradiation was set at -4 ppm, while the off-resonance was set to -40 ppm. Samples contained a concentration of 1 mM of **2**, 1 mM or 10 mM of CTP and 10 µM of PaIspD resulting in a 100:1 ratio regarding **2** and the enzyme. Spectra were processed using Topspin 4.2.0, Bruker's NMR Data Analysis software.

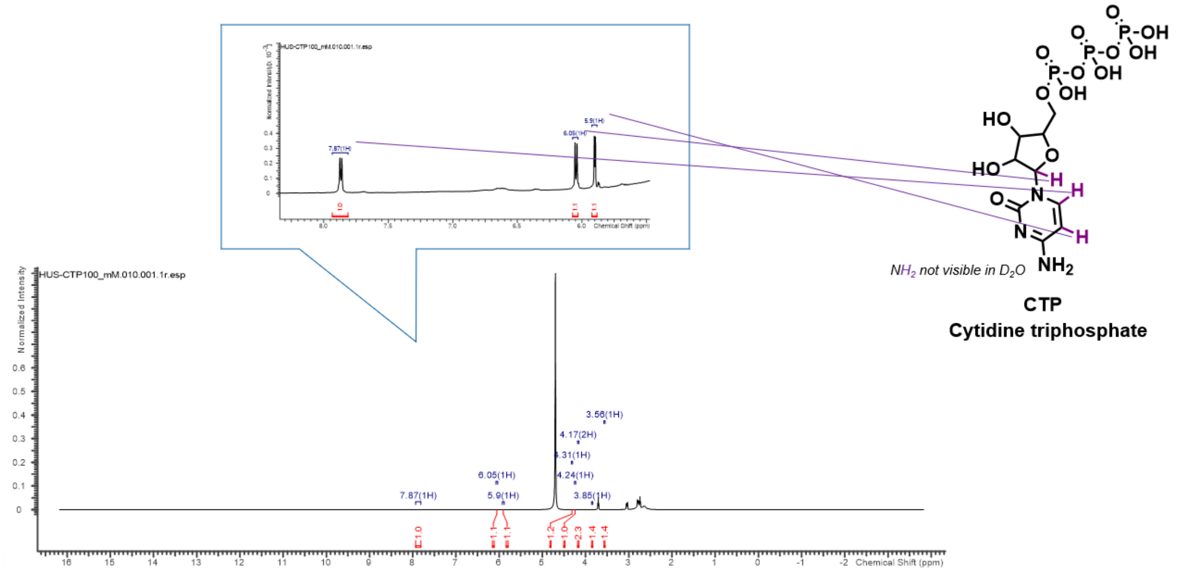

Figure S4. ^{1}H NMR spectrum of CTP at 40 mM in deuterated HEPES buffer

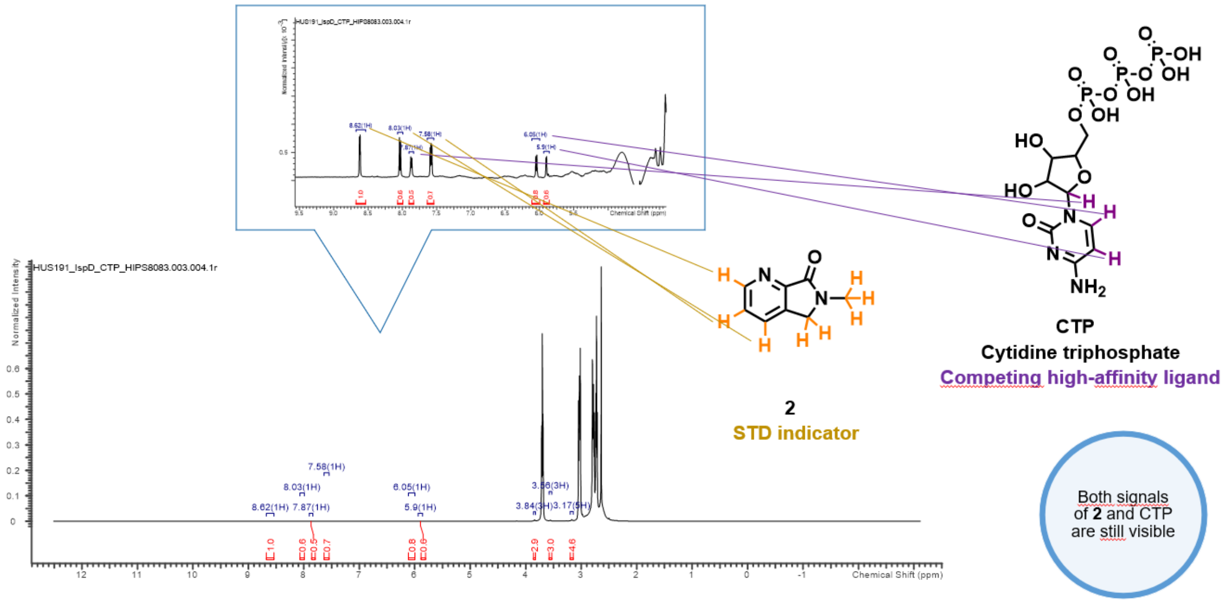

Figure S5. ^{1}H STD-NMR spectrum of **2** used as an STD indicator at 1 mM and CTP used as competing ligand at 1 mM in presence of 10 µM *Pa*IspD.

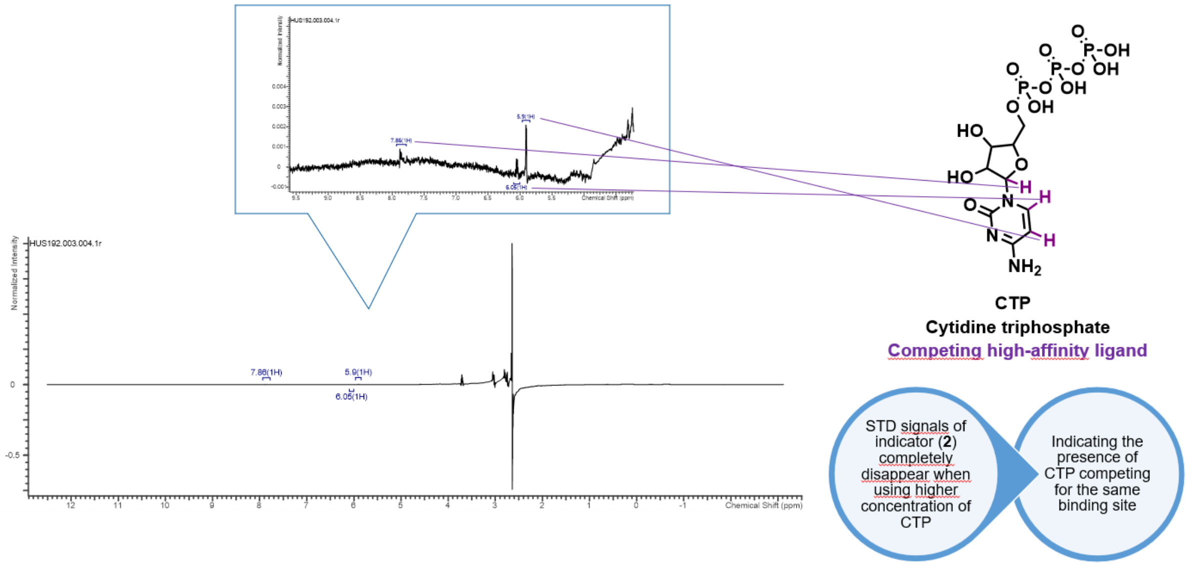

Figure S6. ^{1}H STD-NMR spectrum of **2** used as an STD indicator at 1 mM and CTP used as competing ligand at 10 mM in presence of 10 µM *Pa*IspD

MST Procedure (*Pa*IspD)

The microscale thermophoresis (MST) (Serial no. 201709-BR-N024, Monolith NT.115 Micro Scale Thermophoresis, NanoTemper Technologies GmbH.) was performed using dyed *Pa*IspD, labeled according to the standard protocol from the manufacturer NanoTemper Technologies GmbH Protein Labeling Kit Red-NHS, 2nd Generation. A 2 mM ligand solution containing 10% DMSO was prepared (Solution I). A 200 µl solution of 10% DMSO containing buffer solution was prepared (Solution II). The buffer used was HEPES (50 mM), $MgCl_2$ (5 mM) and Tween (0.05%) at pH 7.6 for all compounds. To PCR tubes 2-16, 10 µl of solution II was added. To tube 1, 20 µl of solution I was added. 10 µl from tube 1 was add to tube 2 and mixed. This was continued until tube 16. To each tube, 10 µL of the protein dye mixture was added and mixed. The mixture was allowed to incubate at room temperature for 5 minutes. Non-hydrophobic capillary tubes were used for measurement. Each sample was measured and analyzed in MO Control version 1.6.

Fluorescence quenching (*Pa*IspD)

Quenching is expressed as (I_0 – I) / I_0 with I_0 the intensity (at 332 nm) of fluorescence in absence of compound and I the intensity of fluorescence upon addition of compound. Inset, fluorescence emission spectra in the presence of increasing concentrations of compound. The concentration of *Pa*IspD was kept at 2 µM during the assay, the compounds were tested in concentration ranges given below. The excitation wavelength was set at 286 nm, emission spectra were recorded between 300 nm and 380 nm. The data were fitted using Qtiplot software (qtiplot.com). A least square fit to a simple bimolecular equilibrium was used to estimate the K_d values. Each measurement was reproduced two times. The measurements were carried out in the following buffer: 50 mM Tris-Cl pH = 8, 50 mM NaCl, 1mM $MgCl_2$.

Table S1. Concentration ranges used to determine Kd during the Fluorescence quenching assay.

	Concentration range (µM)
2	6000->46,9
3	6000->46,9
4	4000->31,2
5	ND
6	5000->39,1
7	ND
8	6000->46,9
9	ND
10	6000->46,9
11	6000->46,9
12	6000->46,9

Chemistry

General chemistry. Starting materials and solvents were purchased from commercial suppliers, and used without further purification. All chemical yields refer to purified compounds and were not optimized. Column chromatography was performed using the automated flash chromatography system CombiFlash®Rf (Teledyne Isco) equipped with RediSepRf silica columns. Preparative RP-HPLC was performed either using an UltiMate 3000 Semi-Preparative System (Thermo Fisher Scientific) equipped with nucleodur®C18 Gravity (250 mm × 16 mm, 5 µm) column or using a Pure C-850 Flash/Prep (Buchi) equipped with Nucleodur C18 HTec (250 mm x 40 mm, particle size 5 µm). Low resolution mass spectrometry and purity control of final compounds was carried out using an Ultimate 3000-MSQ LCMS system (Thermo Fisher Scientific) consisting of a pump, an autosampler, MWD detector and an ESI quadrupole mass spectrometer. ^{1}H and ^{13}C NMR spectra were recorded as indicated on a Bruker Avance Neo 500 MHz (^{1}H, 500 MHz; ^{13}C, 126 MHz) with prodigy cryoprobe system. Chemical shifts were recorded as *δ* values in ppm units and referenced against the residual solvent peak (DMSO-d_6, *δ*= 2.50, 39.52 and $CDCl_3$-*d1*: *δ*=7.26, 77.16). Splitting patterns describe apparent multiplicities and are designated as s (singlet), br s (broad singlet), d (doublet), dd (doublet of doublet), t (triplet), q (quartet), m (multiplet). Coupling constants (*J*) are given in Hertz (Hz). High-resolution mass spectra were recorded on a ThermoFisher Scientific (TF, Dreieich, Germany) Q Exactive Focus system equipped with heated electrospray ionization (HESI)-II source.

General procedure (GP-1): Synthesis of Derivatives 2–6

To a crimp vial, methyl 3-(bromomethyl)picolinate (1.0 equiv), the respective primary amine (1.2 equiv) and THF (175 equiv) were added, the vial was capped off, heated to 80 °C and stirred for 2.5 h. Next water (20 mL) was added and the resulting solution was extracted with a 3/1 mixture of $CHCl_3$ and propanol (5x 15 mL), the combined organic layers were dried over $MgSO_4$, filtered, concentrated *in vacuo*, and purified by preparative HPLC.

General procedure (GP-2): Synthesis of Derivatives 8–11

To a flask were added, 6-methyl-5-(methylamino)-5,6-dihydro-7*H*-pyrrolo[3,4-β]pyridin-7-one (1.0 equiv), the respective carboxylic acid (1.2 equiv), trimethylamine (2 equiv), and DCM (140 equiv). The resulting solution was stirred for 10 min at room temperature, after which, propanephosphonic acid anhydride (1.5 equiv) was added and the resulting mixture was stirred at room temperature overnight. Next, water (20 mL) was added and the resulting solution was extracted with a 3/1 mixture of $CHCl_3$ and propanol (5x 15 mL), organic fractions were dried over $MgSO_4$, filtered, and the solvent was removed *in vacuo*. The residue was purified using preparative HPLC.

6-Methyl-5,6-dihydro-7*H*-pyrrolo[3,4-β]pyridin-7-one (**2**)

According to **GP-1**, using methylamine (0.04 g, 0.48 mmol), afforded after purification by preparative HPLC, using H_2O and ACN as solvent in a gradient (0% to 100% ACN), **2** as a white powder (0.01 g, 18% yield). ^{1}H NMR (500 MHz, DMSO-d_6, ppm) *δ* = 8.69 (d, *J* = 4.6 Hz, 1H), 8.05 (d, *J* = 7.6 Hz, 1H), 7.55 (dd, *J* = 7.7, 4.8 Hz, 1H), 4.48 (s, 2H), 3.12 (s, 3H). ^{13}C NMR (126 MHz, DMSO-d_6, ppm) 165.6, 150.3, 150.0, 136.0, 131.8, 125.1, 49.1, 29.4. HRMS (ESI$^+$) calculated for $C_8H_9N_2O$ [M+H]$^+$ 149.06366, found 149.07069.

6-(1-Methylpiperidin-4-yl)-5,6-dihydro-7*H*-pyrrolo[3,4-β]pyridin-7-one (**3**)

According to **GP-1**, using 4-amino-1-methylpiperidine (0.04 g, 0.26 mmol), afforded after purification by preparative HPLC, **6** as a white powder (0.01 g, 16% yield). ^{1}H NMR (500 MHz, $CDCl_3$-*d*, ppm) *δ* = 8.80 (d, *J* = 4.4 Hz, 1H), 7.88 – 7.84 (m, 1H), 7.46 (dd, *J* = 7.7, 4.8 Hz, 1H), 4.61 (tt, *J* = 12.2, 4.1 Hz, 1H), 4.46 (s, 2H), 3.43 (br d, *J* = 12.1 Hz, 2H), 2.75 – 2.67 (m, 2H), 2.66 (s, 3H), 2.44 (qd, *J* = 12.8, 3.7 Hz, 2H), 1.99 (br dd, *J* = 12.7, 1.5 Hz, 2H). ^{13}C NMR (126 MHz, $CDCl_3$-*d*, ppm) *δ* = 167.6, 166.6, 151.3, 150.7, 135.4, 131.7, 125.6, 54.4, 47.1, 44.4, 44.0, 28.0. HRMS (ESI$^+$) calculated for $C_{13}H_{18}N_3O$ [M+H]$^+$ 232.13716, found 232.14404.

6-Benzyl-5,6-dihydro-7*H*-pyrrolo[3,4-β]pyridin-7-one (**4**)

According to **GP-1**, using benzylamine (0.03 g, 0.26 mmol), afforded after purification by preparative HPLC **3** as a white powder (0.02 g, 48% yield).^{1}H NMR (500 MHz, $CDCl_3$-*d*, ppm) *δ* = 8.81 (br s, 1H), 7.84 – 7.70 (m, 1H), 7.43 (br s, 1H), 7.38 – 7.28 (m, 5H), 4.88 (s, 2H), 4.28 (br s, 2H). ^{13}C NMR (126 MHz, $CDCl_3$-*d*, ppm) *δ* = 165.8, 150.3, 150.2, 136.3, 135.4, 131.9, 128.9, 128.4, 128.0, 125.4, 47.2, 46.9. HRMS (ESI$^+$) calculated for $C_{14}H_{13}N_2O$ [M+H]$^+$ 225.09496, found 225.10170.

6-(2-Morpholinoethyl)-5,6-dihydro-7*H*-pyrrolo[3,4-β]pyridin-7-one (**5**)

According to **GP-1**, using 4-(2-aminoethyl)morpholine (0.03 g, 0.26 mmol), afforded after purification by preparative HPLC **5** as a white powder (0.01 g, 18% yield). ^{1}H NMR (500 MHz, $CDCl_3$-*d*, ppm) δ = 8.80 (d, *J* = 4.7 Hz, 1H), 7.85 (d, *J* = 7.6 Hz, 1H), 7.45 (dd, *J* = 7.7, 4.8 Hz, 1H), 4.61 (s, 2H), 3.95 (br s, 2H), 3.81 (br s, 4H), 2.90 (br s, 2H), 2.75 (br s, 4H). ^{13}C NMR (126 MHz, $CDCl_3$-*d*, ppm) δ = 166.6, 150.6, 150.3, 135.1, 130.9, 125.0, 66.0, 56.0, 53.0, 48.4, 38.8. HRMS (ESI$^+$) calculated for $C_{13}H_{18}N_3O_2$ [M+H]$^+$ 248.13208, found 248.13901.

6-(2-(1*H*-Indol-3-yl)ethyl)-5,6-dihydro-7*H*-pyrrolo[3,4-β]pyridin-7-one (**6**)

According to **GP-1**, using tryptamine (0.04 g, 0.26 mmol), afforded after purification by preparative HPLC **4** as a white solid (0.01g, 23% yield). ^{1}H NMR (500 MHz, $CDCl_3$-*d*, ppm) *δ* = 8.78 (d, *J* = 4.7 Hz, 1H), 8.15 (br s, 1H), 7.71 (d, *J* = 7.8 Hz, 1H), 7.63 (d, *J* = 7.8 Hz, 1H), 7.43 – 7.34 (m, 2H), 7.23 – 7.18 (m, 1H), 7.14 – 7.08 (m, 2H), 4.22 (s, 2H), 4.08 (t, *J* = 6.9 Hz, 2H), 3.22 (t, *J* = 6.9 Hz, 2H). ^{13}C NMR (126 MHz, $CDCl_3$-*d*, ppm) δ = 166.5, 151.0, 150.7, 136.3, 135.0, 131.0, 127.2, 124.9, 119.5, 118.5, 112.5, 111.3, 48.2, 43.1, 24.3. HRMS (ESI$^+$) calculated for $C_{17}H_{16}N_3O$ $[M+H]^+$ 278.12151, found 278.12811.

6-Methyl-5-(methylamino)-5,6-dihydro-7*H*-pyrrolo[3,4-β]pyridin-7-one (**I**)

To a crimp vial, methyl 3-methylpicolinate (1.0 g, 6.6 mmol), 1-bromopyrrolidine-2,5-dione (2.60 g, 14.6 mmol),*di*benzoylperoxide (0.01 g, 0.2 mmol), and $CHCl_3$ (10 mL) were added. The vial was sealed and stirred at 80 °C overnight, after which, water (20 mL) was added and the resulting solution was extracted with CH_2Cl_2 (3x, 15 mL). The combined organic layers were dried over $MgSO_4$, filtered, concentrated *in vacuo*, and purified by flash chromatography using CH_2Cl_2 as solvent. Then, the combined fractions were concentrated *in vacuo* and resolubilized in water (50 mL) to which an excess of methylamine (40% w/v water) was added and, the resulting mixture was stirred at 80 °C overnight. The reaction mixture was extracted using a 3/1 mixture of $CHCl_3$ and propanol (5x, 15 mL), combined organic layers were dried over $MgSO_4$, filtered, and concentrated *in vacuo* to afford **I** as an off-white solid (0.74 g, 63% crude yield). ^{1}H NMR (500 MHz, $CDCl_3$-*d*, ppm) *δ* = 8.82 – 8.78 (m, 1H), 7.94 – 7.86 (m, 1H), 7.49 – 7.42 (m, 1H), 5.33 (s, 1H), 3.16 – 3.10 (m, 3H), 2.01 (s, 3H).

Methyl methyl(6-methyl-7-oxo-6,7-dihydro-5*H*-pyrrolo[3,4-β]pyridin-5-yl)carbamate (**7**).

To a flask containing, **I** (0.05 g, 0.28 mmol), trimethylamine (0.06 g, 0.62 mmol), and DMF (2 mL), methylchloroformate (0.04 g, 0.42 mmol) was added at 0 °C. The resulting solution was stirred at 0 °C for 2 h, after which, water (15 mL) was added and the resulting solution was extracted using a 3/1 mixture of $CHCl_3$ and propanol (5x, 15 mL), combined organic layers were dried over $MgSO_4$, filtered, concentrated *in vacuo*, and purified by preparative HPLC, affording **7** as a white solid (0.02 g, 33% yield). ^{1}H NMR (500 MHz, $CDCl_3$-*d*, ppm) *δ* = 8.85 (br d, *J* = 4.1 Hz, 1H), 7.80 (br d, *J* = 7.8 Hz, 1H), 7.53 – 7.44 (m, *J* = 5.0 Hz, 1H), 6.76 (s, 1H), 3.96 – 3.81 (m, 3H), 3.08 (s, 3H), 2.45 – 2.31 (m, 3H). ^{13}C NMR (126 MHz, $CDCl_3$-*d*) δ = 166.1, 158.0, 152.6, 151.6, 135.1, 131.4, 126.0, 69.5, 53.8, 27.5, 27.1. HRMS (ESI$^+$) calculated for $C_{11}H_{14}N_3O_3$ $[M+H]^+$ 236.09569, found 236.10240.

***N*-Methyl-*N*-(6-methyl-7-oxo-6,7-dihydro-5*H*-pyrrolo[3,4-β]pyridin-5-yl)propionamide** (**8**).

According to **GP-2**, using propionic acid (0.03 g, 0.34 mmol), afforded after purification by preparative HPLC, **8** as a white powder (0.03 g, 45% yield). ^{1}H NMR (500 MHz, $CDCl_3$-*d*, ppm) *δ* = 8.83 (d, *J* = 4.7 Hz, 1H), 7.75 (d, *J* = 7.6 Hz, 1H), 7.47 (dd, *J* = 7.7, 4.8 Hz, 1H), 7.19 (s, 1H), 3.09 – 3.02 (m, 3H), 2.59 – 2.46 (m, 2H), 2.44 (s, 3H), 1.26 (t, *J* = 7.4 Hz, 3H). ^{13}C NMR (126 MHz, $CDCl_3$-*d*, ppm) *δ* = 176.0, 166.1, 152.2, 151.5, 135.2, 131.1, 125.7, 66.4, 28.1, 27.4, 26.9, 9.0. HRMS (ESI$^+$) calculated for $C_{12}H_{16}N_3O_2$ $[M+H]^+$ 234.11643, found 234.12293.

2-Ethyl-*N*-methyl-*N*-(6-methyl-7-oxo-6,7-dihydro-5*H*-pyrrolo[3,4-β]pyridin-5-yl)butanamide (**9**).

According to **GP-2**, using 2-ethylbutanoic acid (0.04 g, 0.34 mmol), afforded after purification by preparative HPLC, **9** as a white powder (0.01 g, 4% yield). ^{1}H NMR (500 MHz, $CDCl_3$-*d*, ppm) *δ* = 8.87 – 8.83 (m, 1H), 7.71 (d, *J* = 7.3 Hz, 1H), 7.48 (dd, *J* = 7.6, 4.9 Hz, 1H), 7.06 (s, 1H), 3.11 – 3.04 (m, 3H), 2.69 – 2.62 (m, 1H), 2.49 (m, 3H), 1.85 – 1.73 (m, 2H), 1.68 – 1.56 (m, 2H), 1.03 – 0.94 (m, 6H). ^{13}C NMR (126 MHz, $CDCl_3$-d) *δ* = 178.5, 166.1, 152.2, 151.6, 135.4, 131.0, 125.8, 66.4, 45.7, 28.4, 27.0, 26.0, 25.7, 12.1. HRMS (ESI$^+$) calculated for $C_{15}H_{22}N_3O_2$ $[M+H]^+$ 276.16338, found 276.17015.

***N*-Methyl-*N*-(6-methyl-7-oxo-6,7-dihydro-5*H*-pyrrolo[3,4-β]pyridin-5-yl)isonicotinamide** (**10**).

According to **GP-2**, using isonicotinic acid (0.04 g, 0.34 mmol), afforded after purification by preparative HPLC, **10** as a white powder (0.04 g, 45% yield). ^{1}H NMR (500 MHz, $CDCl_3$-*d*, ppm) *δ* = 8.90 (d, *J* = 4.6 Hz, 1H), 8.80 (br d, *J* = 4.4 Hz, 2H), 7.92 (br d, *J* = 7.6 Hz, 1H), 7.59 – 7.51 (m, 1H), 7.47 – 7.37 (m, 2H), 7.21 (s, 1H), 3.23 – 3.04 (m, 2H), 2.62 – 2.38 (m, 2H). ^{13}C NMR (126 MHz, $CDCl_3$-*d*, ppm) *δ* = 171.6, 166.3, 152.9, 150.9, 142.4, 134.7, 131.4, 126.3, 121.3, 67.1, 30.7, 27.5. HRMS (ESI$^+$) calculated for $C_{15}H_{15}N_4O_2$ $[M+H]^+$ 283.11168, found 283.11870.

***N*-Methyl-*N*-(6-methyl-7-oxo-6,7-dihydro-5*H*-pyrrolo[3,4-β]pyridin-5-yl)isoxazole-3-carboxamide** (**11**).

According to GP-2, using isoxazole-3-carboxylic acid (0.04 g, 0.34 mmol), afforded after purification by preparative HPLC, **11** as a white powder (0.06, 75% yield). ^{1}H NMR (500 MHz, DMSO-*d$_6$*) *δ* = 9.21 (s, 1H), 8.83 – 8.82 (m, 1H), 8.20 – 8.15 (m, 1H), 7.71 – 7.67 (m, 1H), 7.07 (s, 1H), 7.02 (s, 1H), 6.57 – 6.51 (m, 1H), 2.92 (s, 3H), 2.46 (s, 3H). ^{13}C NMR (126 MHz, DMSO-*d$_6$*, ppm) *δ* = 161.6, 161.2, 160.7, 157.0, 151.9, 134.9, 131.7, 126.2, 105.4, 70.8, 26.1. HRMS (ESI$^+$) calculated for $C_{13}H_{13}N_4O_3$ $[M+H]^+$ 273.09094, found 273.09814.

***N*-Methyl-*N*-(6-methyl-7-oxo-6,7-dihydro-5*H*-pyrrolo[3,4-β]pyridin-5-yl)morpholine-4-carboxamide (12).**

To a flask at 0 °C containing, **I** (0.05 g, 0.28mmol), trimethylamine (0.06 g, 0.62 mmol), and CH_2Cl_2 (3 mL), morpholine-4-carbonyl chloride (0.06 g, 0.42 mmol) was added. The resulting solution was stirred at 0 °C for 2 h, after which, water (15 mL) was added and the resulting solution was extracted using a 3/1 mixture of $CHCl_3$ and propanol (5x, 15 mL), combined organic layers were dried over $MgSO_4$, filtered, concentrated *in vacuo,* and purified by preparative HPLC, affording **12** as

a gray solid (0.02 g, 26% yield). ^{1}H NMR (500 MHz, $CDCl_3$-*d*, ppm) *δ* = 8.84 (d, *J* = 4.0 Hz, 1H), 7.90 (d, *J* = 7.6 Hz, 1H), 7.47 (dd, *J* = 7.7, 4.8 Hz, 1H), 6.45 (s, 1H), 3.79 – 3.74 (m, 4H), 3.45 – 3.37 (m, 4H), 3.13 (s, 3H), 2.38 – 2.33 (m, 3H). ^{13}C NMR (126 MHz, $CDCl_3$-*d*, ppm) δ = 166.2, 164.0, 152.3, 151.4, 135.7, 131.8, 125.9, 70.3, 66.5, 47.2, 30.6, 27.2. HRMS (ESI^+) calculated for $C_{14}H_{19}N_4O_3$ $[M+H]^+$ 291.13789, found 291.14486.

Figures, Schemes and Tables

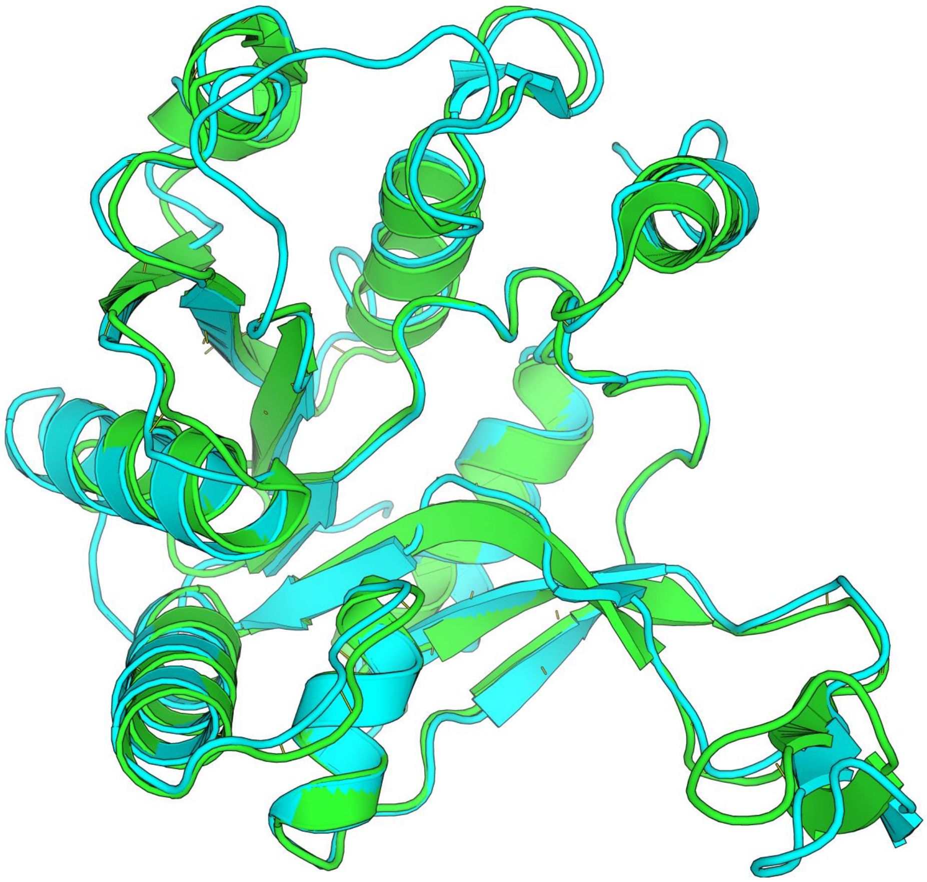

Figure S7. Overlay of the *apo* crystal structures of both *Pseudomonas aeruginosa* and *Escherichia coli* IspD (Protein Data Bank entry 1INJ); Cyan: *Pa*IspD; green: *Ec*IspD

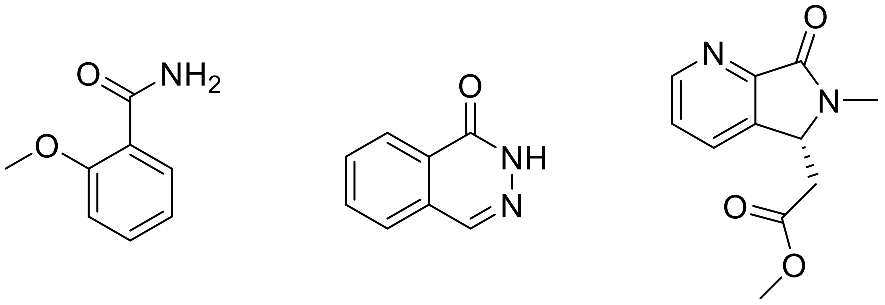

1

Figure S8. Chemical structures of the fragments found during the crystallographic screening.

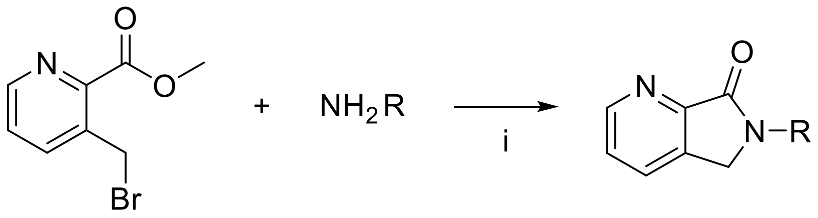

Scheme S1. Synthetic route for the synthesis of **2** – **6**. Reagents and conditions: (i) methyl 3-(bromomethyl)picolinate, the respective primary amine, THF, 80 °C, 2.5 h, 16–48% yield.

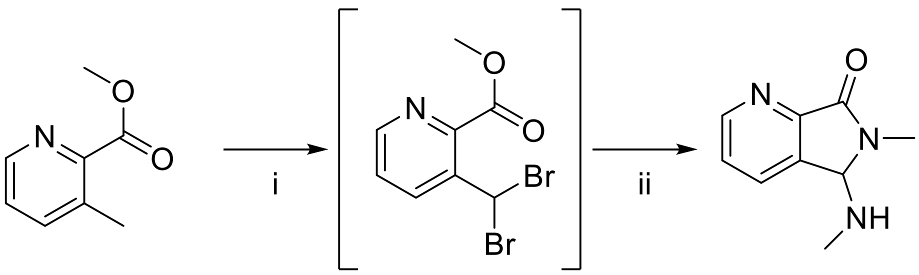

I

Scheme S2. Synthetic route for the synthesis of **I**. Reagents and conditions: (i) methyl 3-methylpicolinate, 1-bromopyrrolidine-2,5-dione, dibenzoylperoxide, $CHCl_3$, 80 °C, overnight. (ii) methylamine (40% w/v water), water, 80 °C, overnight, 64% crude yield.

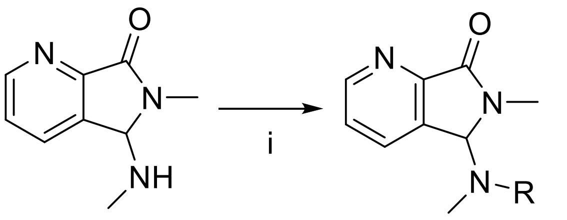

I

Scheme S3. Synthetic route for the synthesis of **7** and **12**. Reagents and conditions: (i) 6-methyl-5-(methylamino)-5,6-dihydro-7*H*-pyrrolo[3,4-β]pyridin-7-one (**I**), the respective acid chloride, trimethylamine, DMF (**7**) / CH_2Cl_2 (**12**) , 0 °C, 2 h, 26–33% yield.

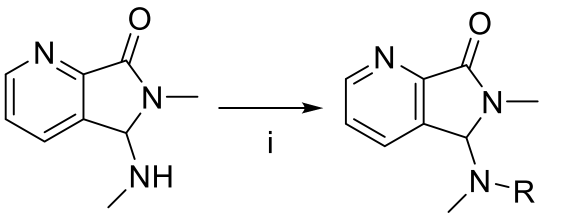

I

Scheme S4. Synthetic route for the synthesis of **8**–**11**. Reagents and conditions: (i) 6-methyl-5-(methylamino)-5,6-dihydro-7*H*-pyrrolo[3,4-β]pyridin-7-one (**I**), the respective carboxylic acid, trimethylamine, propanephosphonic acid anhydride, CH_2Cl_2, room temperature, overnight, 4–75% yield.

Table S2. Data collection and refinement statistics.

	Pa-IspD 1	*Pa*-IspD 2	*Pa*-IspD "apo"
**Crystallization**			
Crystallization condition	26% PEG 400<br>100 mM MES pH 6.5	26% PEG 500MME<br>100 mM MES pH 6.5	26% PEG 500MME 100 mM MES pH 6.5
Ligand	**1**	**2**	none
**Data collection**			
X-ray Source	BL13 XALOC (Alba)	ID30A-3 (ESRF)	ID23-1 (ESRF)
wavelength Å	0.979	0.968	0.885
Resolution (last shell) (Å)	44.70-1.95 (2.02 -	44.2 -2.17 (2.248 - 2.17)	33.2- 1.50 (1.54-1.5)
Total reflections	60026 (6231)	45866 (4132)	217438 (14438)
Unique reflections	18161 (1818)	12788 (2965)	38206 (2549)
Multiplicity (last shell)	3.3 (3.4)	3.6 (3.3)	5.7 (5.7)
Completeness (last shell) (%)	98.78 (98.96)	95.98 (94.47)	98.47 (92.65)
R-merge (last shell) (%)	5.04 (110.2)	6.13 (89.74)	0.03989 (0.9433)
Mean I/σ (last shell)	10.93 (1.27)	11.23 (1.05)	15.01 (1.12)
Space group	C2	C2	C2
Cell parameters			
Dimensions *a, b,c* (Å)	92.15, 75.63, 37.63	91.41, 75.59, 37.8	90,57, 75.37, 37.43
Angle (°)	β = 104.04	β = 104.76	β = 104.80
**Refinement**			
Reflections in refinement	18111 (1807)	12711 (1229)	38184 (2545)
R-work (last shell) (%)	19.88 (35.54)	0.2003 (0.5580)	0.1871 (0.3867)
R-free (last shell) (%)	22.72 (37.48)	23.50 (53.70)	0.2118 (0.4059)
R.m.s.d bonds (Å)	0.004	0.003	0.006
R.m.s.d angles (°)	0.63	0.57	0.88
Average B factor (Å^2)	56.93	56.29	34.00
Water molecules	62	50	169
Ramachandran plot quality (%)			
Most favoured	97.36	97.36	97.36
Additionally allowed	2.64	2.64	2.64

Electron density maps of compounds 4, 5, 8, 10 and 12

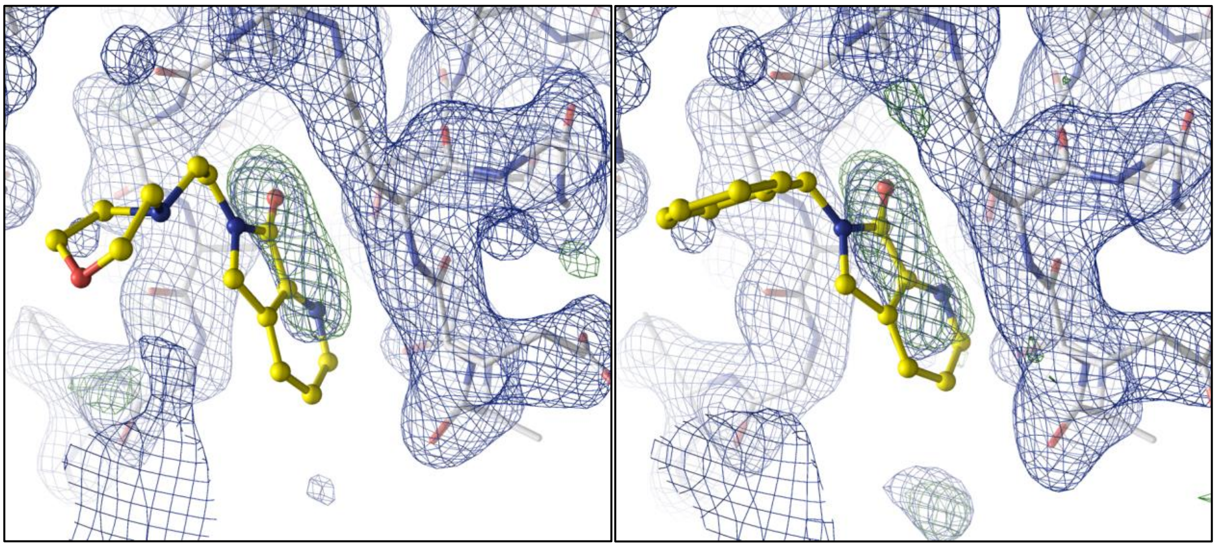

Figure S9. Electron density map of **4** (left) and **5** (right) with *Pa*IspD. Only partial density was seen for the compounds.

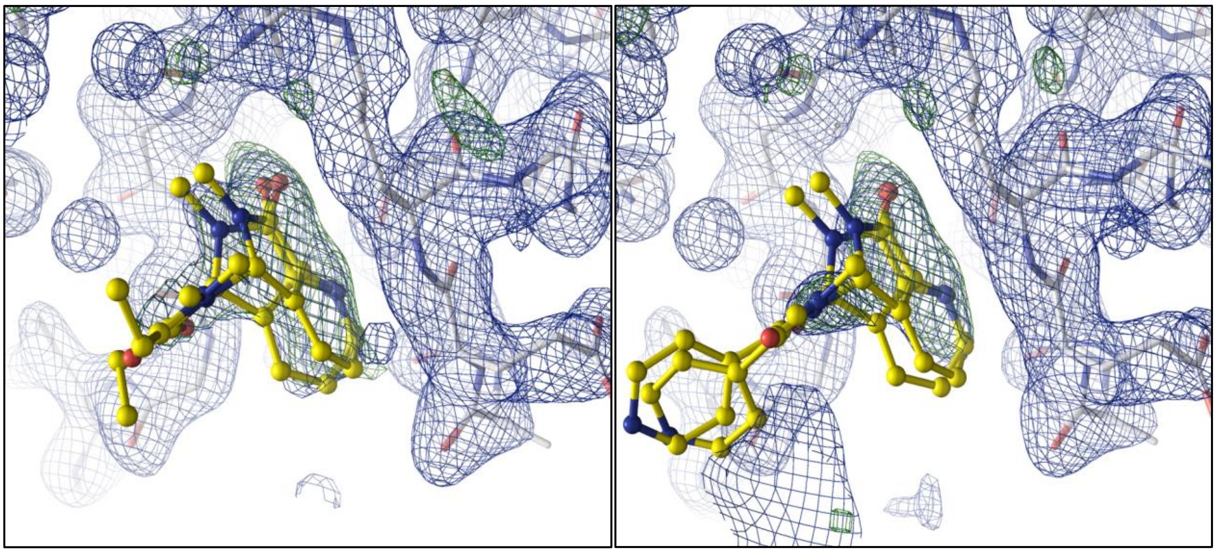

Figure S10. Electron density map of **8** (left) and **10** (right) with *Pa*IspD. Only partial density was seen for the compounds.

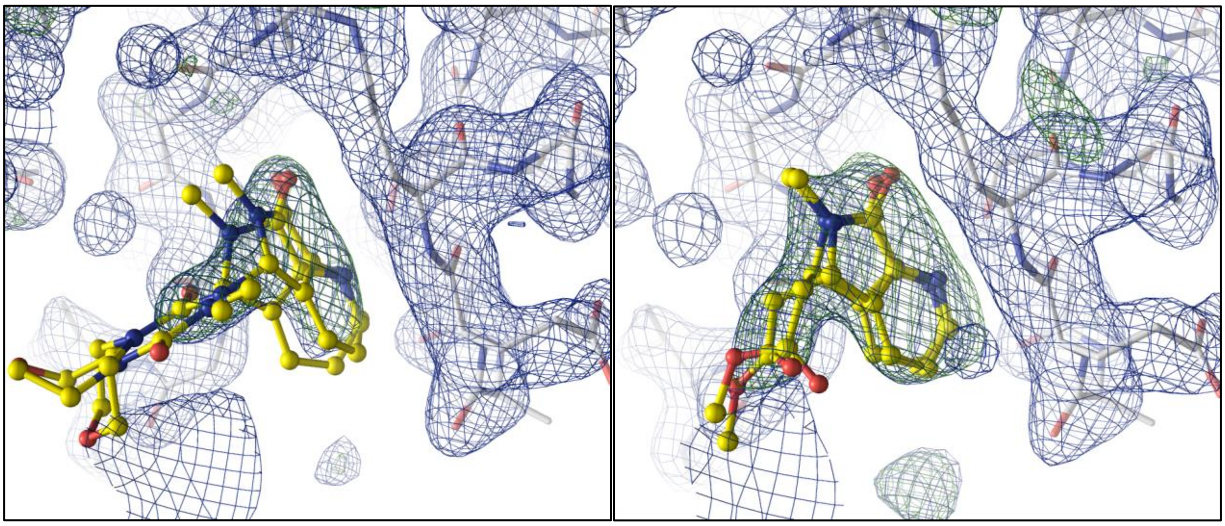

Figure S11. Electron density map of **12** and **1** with *Pa*IspD. Only partial density was seen for the compound **12**.

^{1}H NMR, ^{13}C NMR and LC-MS Spectra of Final Compounds

2

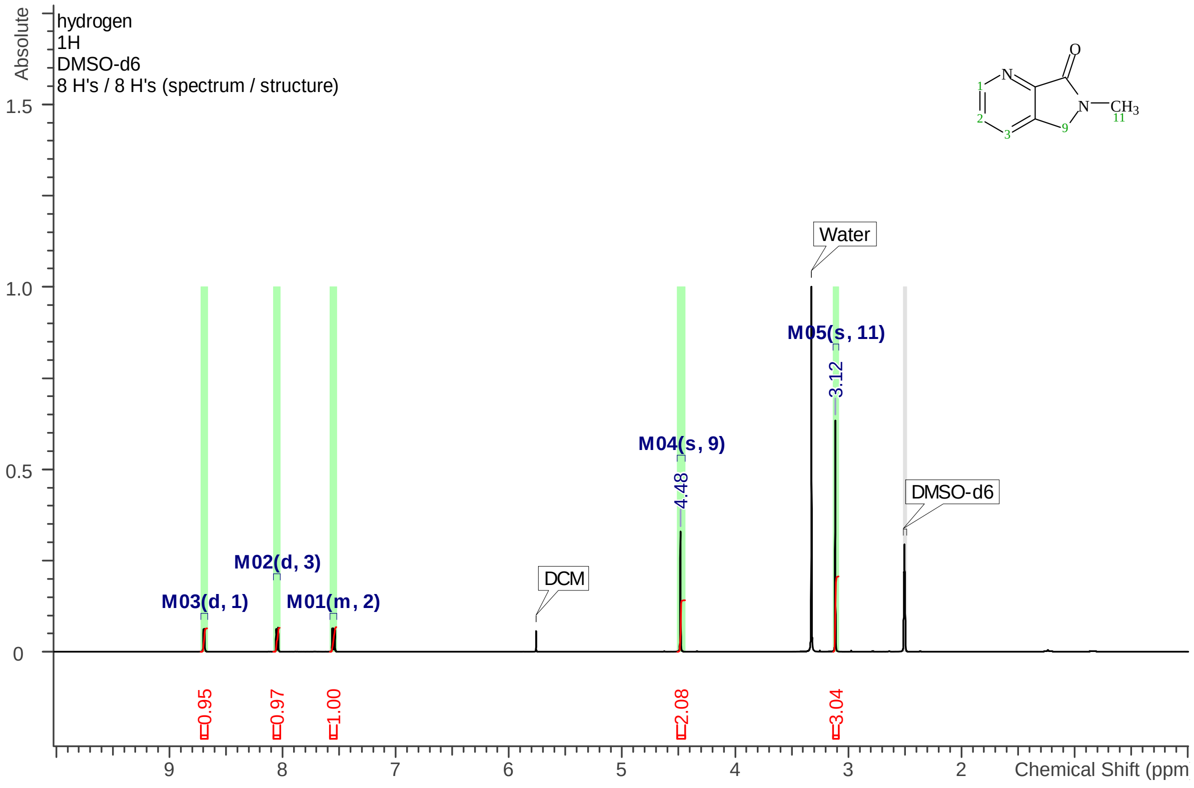

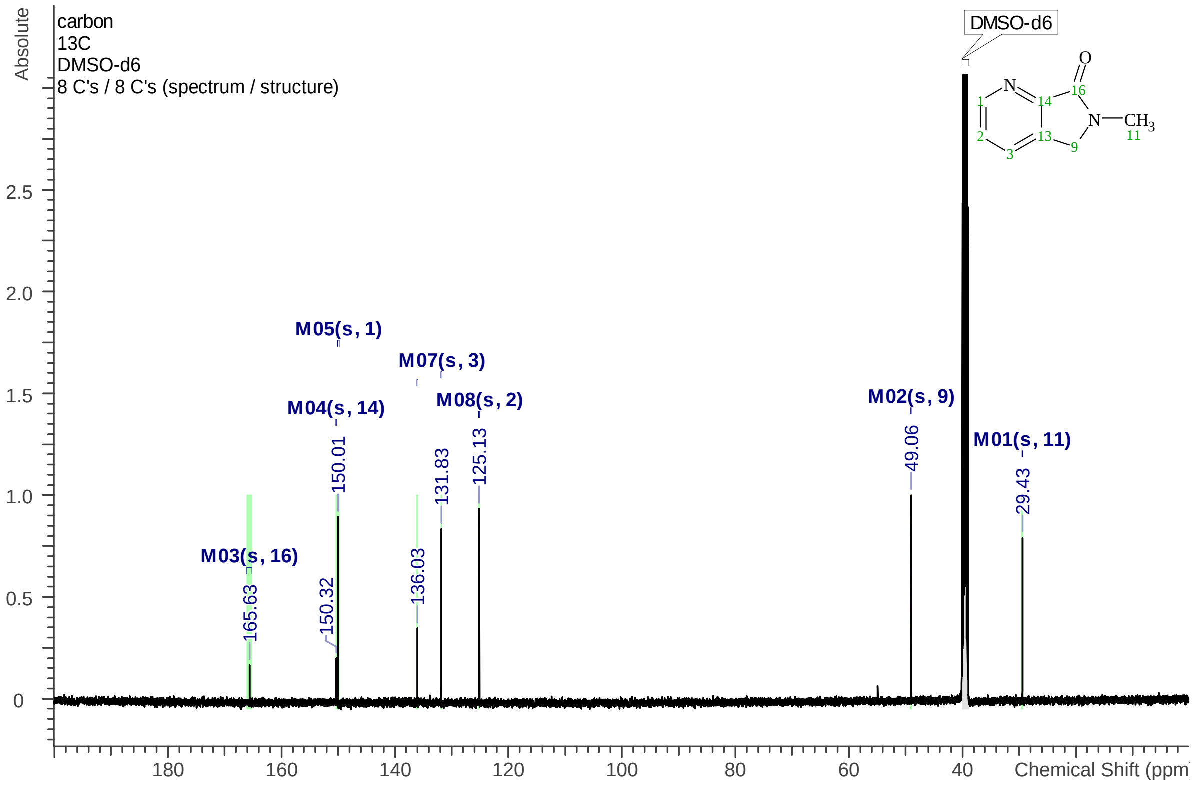

3

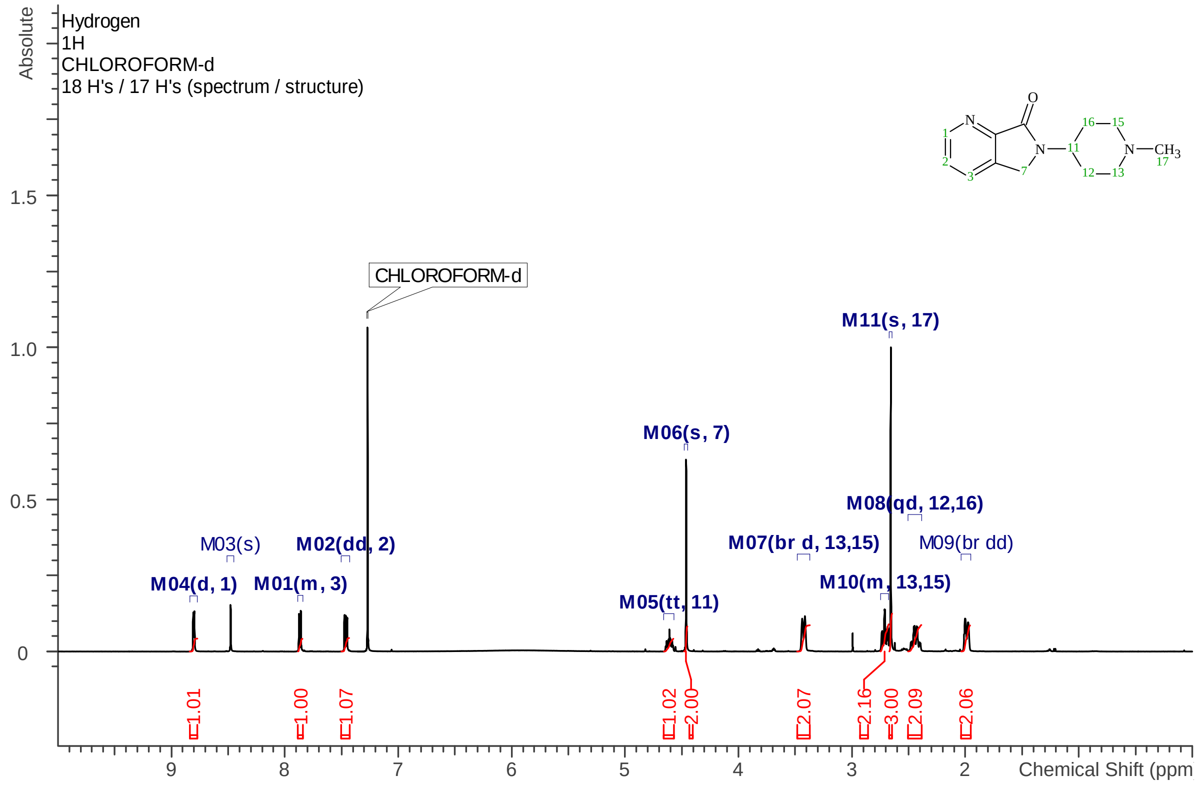
Hydrogen
1H
CHLOROFORM-d
18 H's / 17 H's (spectrum / structure)
CHLOROFORM-d
M11(s, 17)
M06(s, 7)
M08(qd, 12,16)
M03(s)
M02(dd, 2)
M07(br d, 13,15)
M09(br dd)
M04(d, 1)
M01(m, 3)
M10(m, 13,15)
M05(tt, 11)
1.01
1.00
1.07
1.02
2.00
2.07
2.16
3.00
2.09
2.06
Chemical Shift (ppm
Absolute

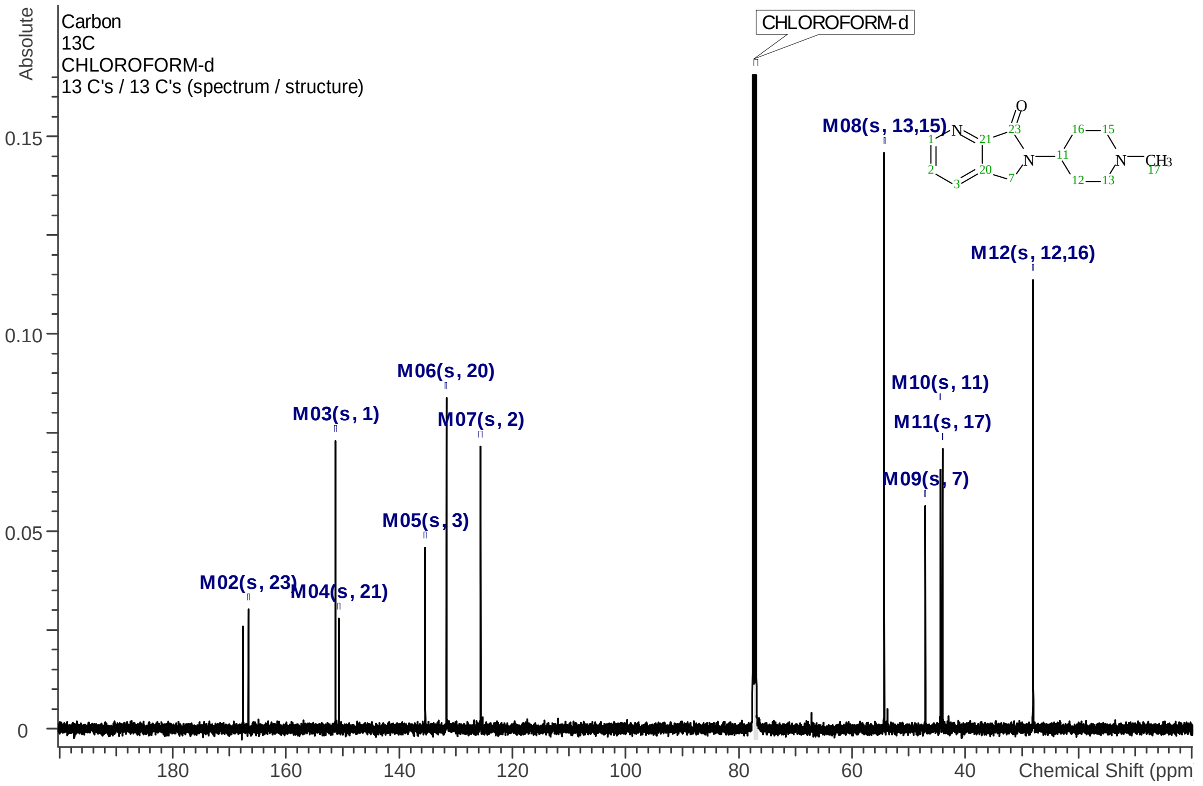
Carbon
13C
CHLOROFORM-d
13 C's / 13 C's (spectrum / structure)
CHLOROFORM-d
M08(s, 13,15)
M12(s, 12,16)
M06(s, 20)
M10(s, 11)
M03(s, 1)
M07(s, 2)
M11(s, 17)
M09(s, 7)
M05(s, 3)
M02(s, 23)
M04(s, 21)
Chemical Shift (ppm
Absolute

4

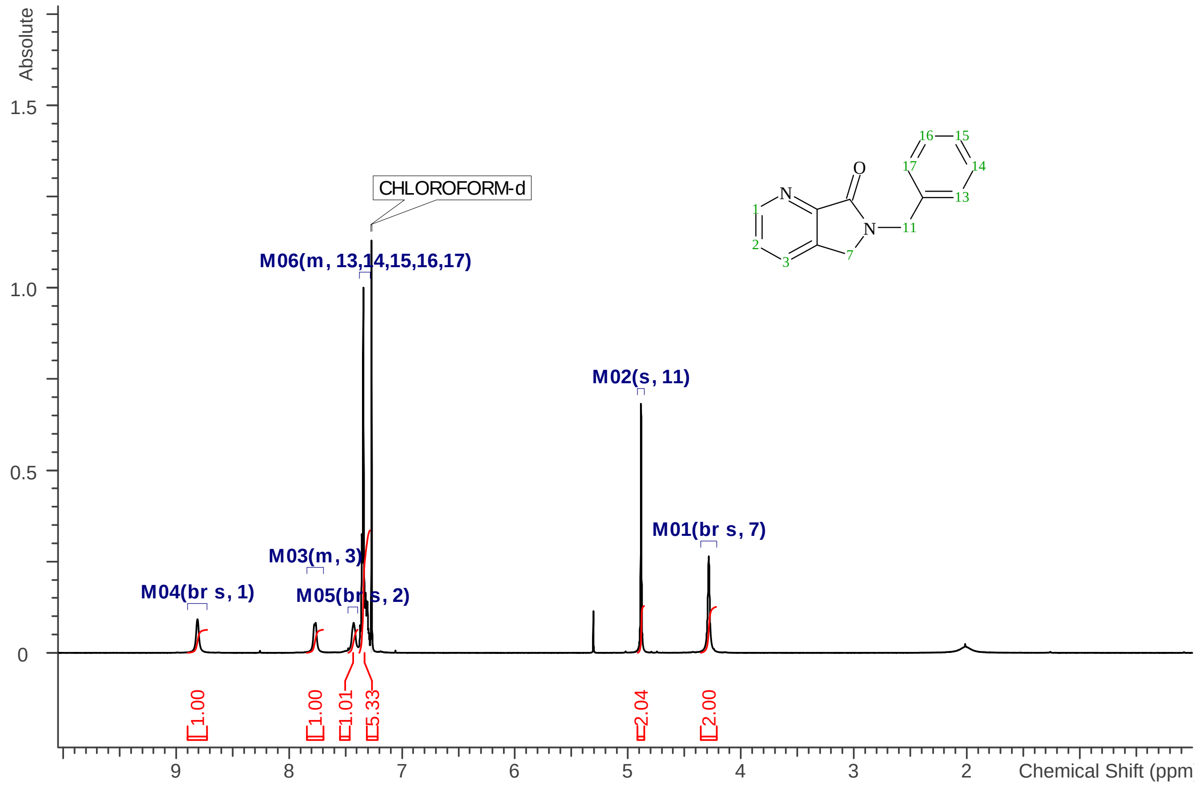

Absolute
CHLOROFORM-d
M06(m, 13,14,15,16,17)
M02(s, 11)
M01(br s, 7)
M03(m, 3)
M04(br s, 1)
M05(br s, 2)
1.00
1.00
1.01
5.33
2.04
2.00
Chemical Shift (ppm

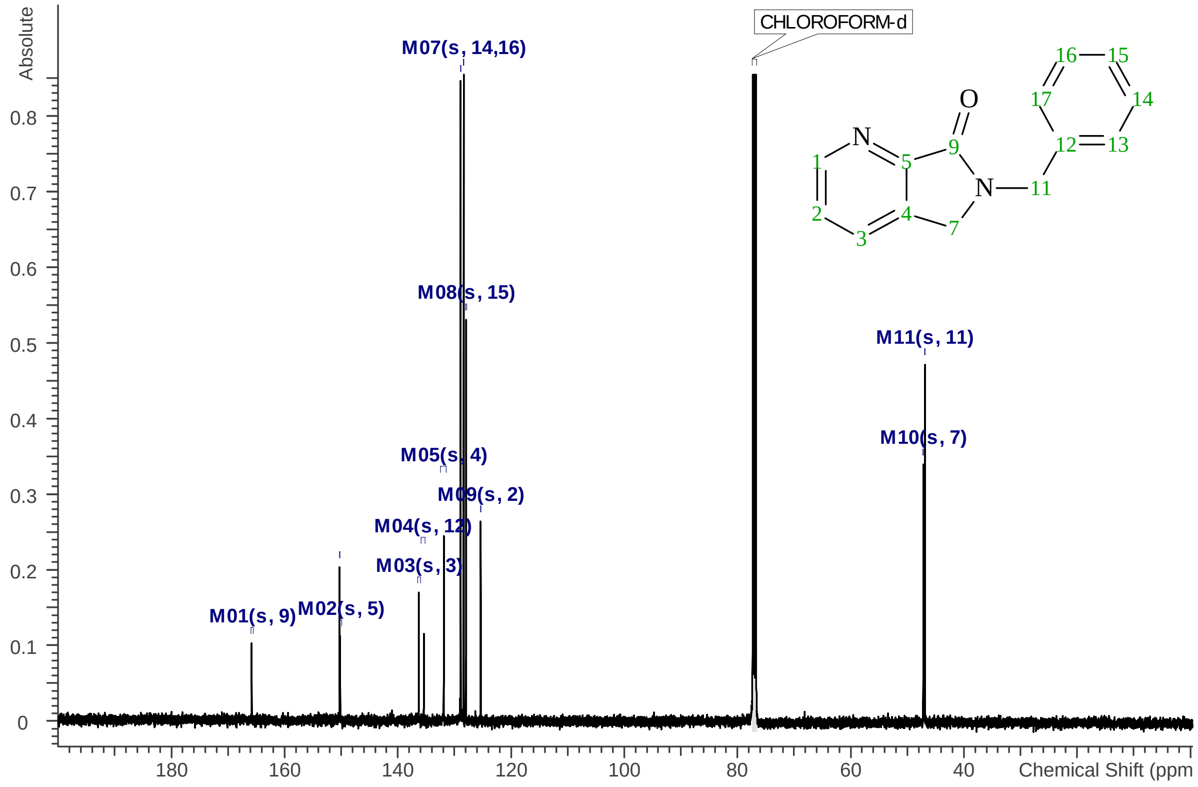

Absolute
CHLOROFORM-d
M07(s, 14,16)
M08(s, 15)
M11(s, 11)
M10(s, 7)
M05(s, 4)
M09(s, 2)
M04(s, 12)
M03(s, 3)
M01(s, 9)
M02(s, 5)
Chemical Shift (ppm

5

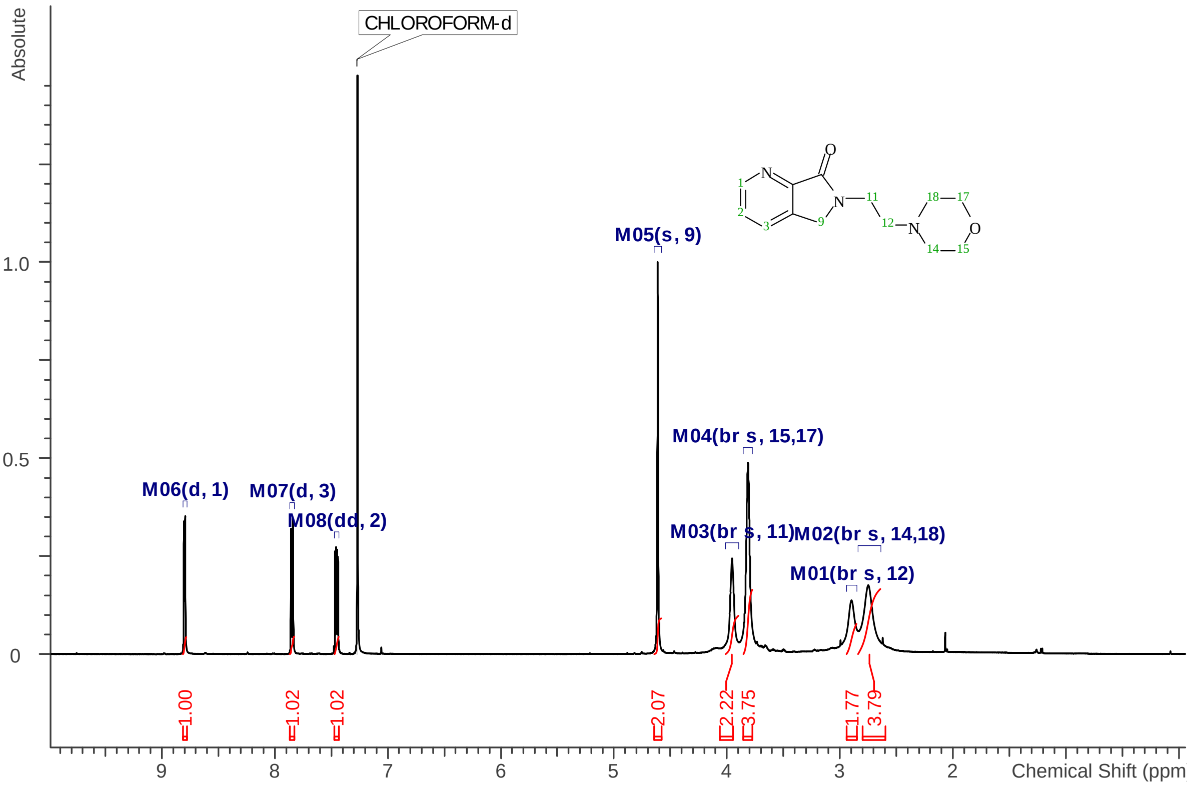

Absolute
CHLOROFORM-d
M05(s, 9)
M04(br s, 15,17)
M06(d, 1)
M07(d, 3)
M08(dd, 2)
M03(br s, 11)
M02(br s, 14,18)
M01(br s, 12)
1.00
1.02
1.02
2.07
2.22
3.75
1.77
3.79
1.0
0.5
0
9
8
7
6
5
4
3
2
Chemical Shift (ppm

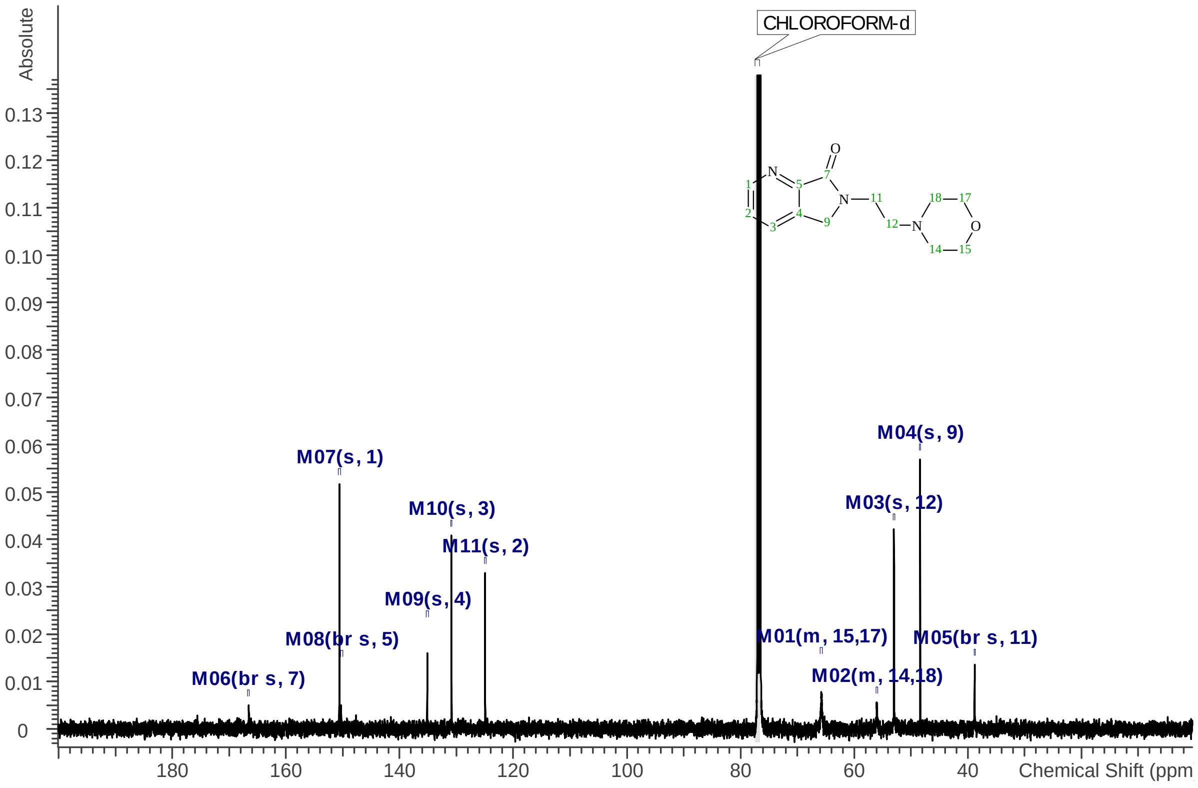

Absolute
CHLOROFORM-d
0.13
0.12
0.11
0.10
0.09
0.08
0.07
0.06
0.05
0.04
0.03
0.02
0.01
0
M07(s, 1)
M10(s, 3)
M11(s, 2)
M09(s, 4)
M08(br s, 5)
M06(br s, 7)
M04(s, 9)
M03(s, 12)
M01(m, 15,17)
M05(br s, 11)
M02(m, 14,18)
180
160
140
120
100
80
60
40
Chemical Shift (ppm

6

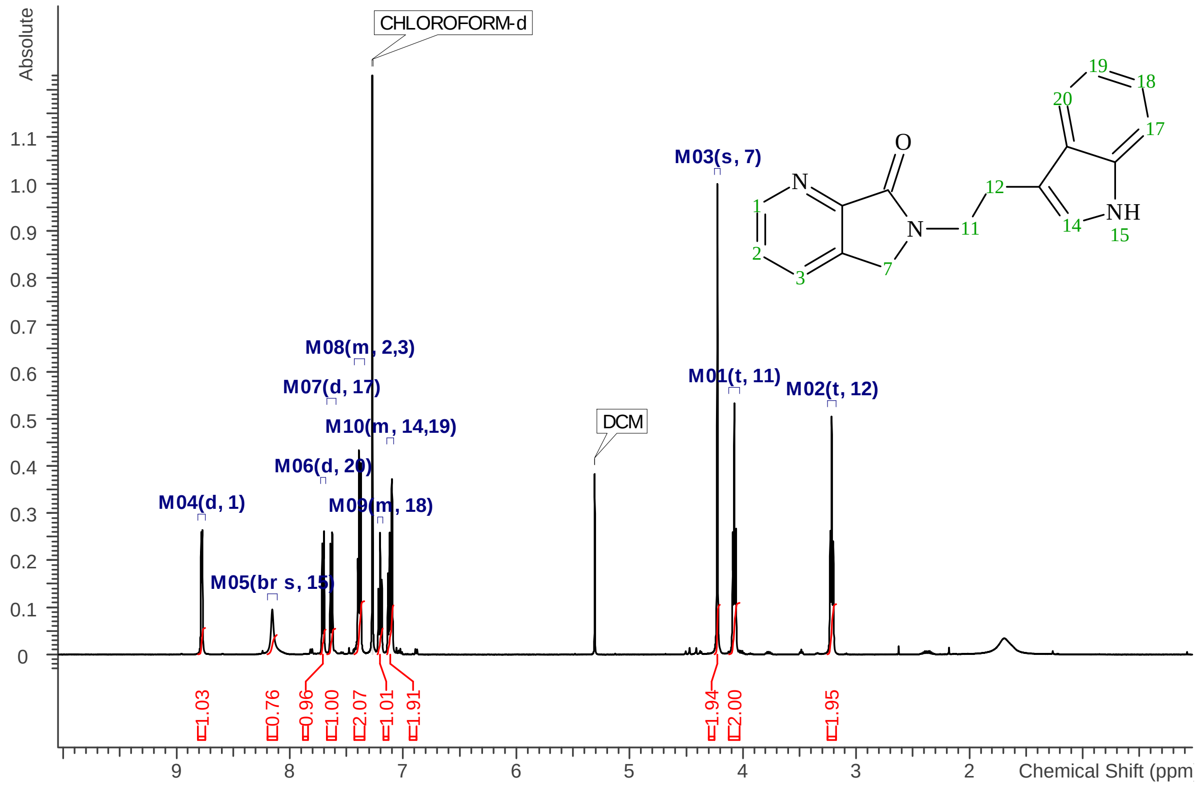
CHLOROFORM-d
DCM
M04(d, 1)
M05(br s, 15)
M06(d, 20)
M07(d, 17)
M08(m, 2,3)
M09(m, 18)
M10(m, 14,19)
M03(s, 7)
M01(t, 11)
M02(t, 12)
1.03
0.76
0.96
1.00
2.07
1.01
1.91
1.94
2.00
1.95
Absolute
Chemical Shift (ppm

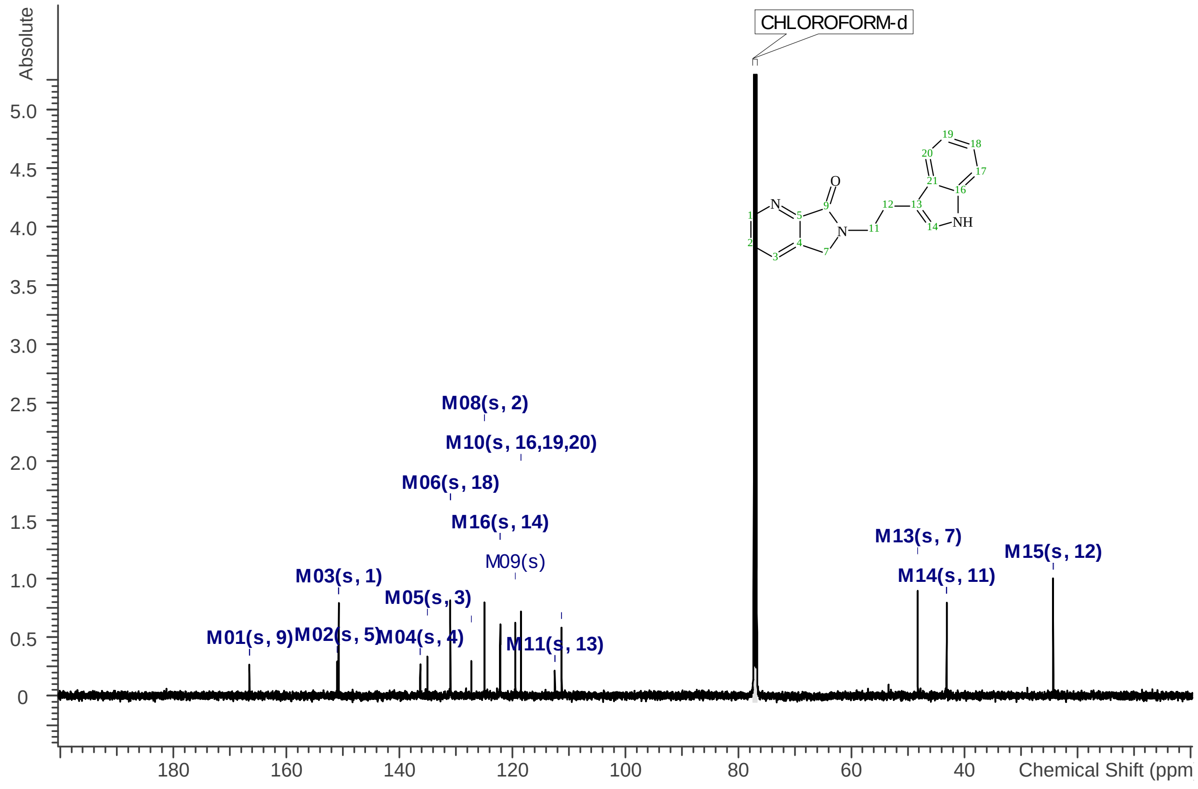
CHLOROFORM-d
M08(s, 2)
M10(s, 16,19,20)
M06(s, 18)
M16(s, 14)
M09(s)
M03(s, 1)
M05(s, 3)
M01(s, 9)
M02(s, 5)
M04(s, 4)
M11(s, 13)
M13(s, 7)
M14(s, 11)
M15(s, 12)
Absolute
Chemical Shift (ppm

7

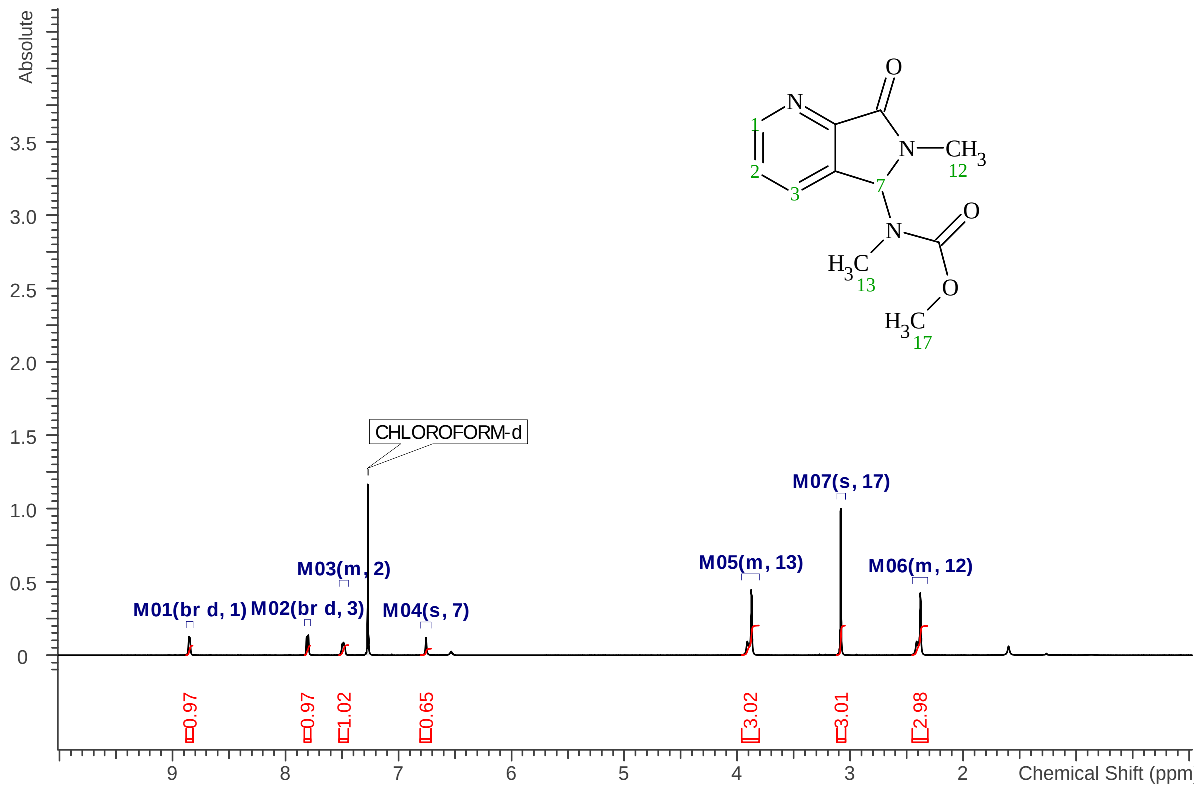
Absolute
CHLOROFORM-d
M01(br d, 1)
M02(br d, 3)
M03(m, 2)
M04(s, 7)
M05(m, 13)
M07(s, 17)
M06(m, 12)
0.97
0.97
1.02
0.65
3.02
3.01
2.98
Chemical Shift (ppm

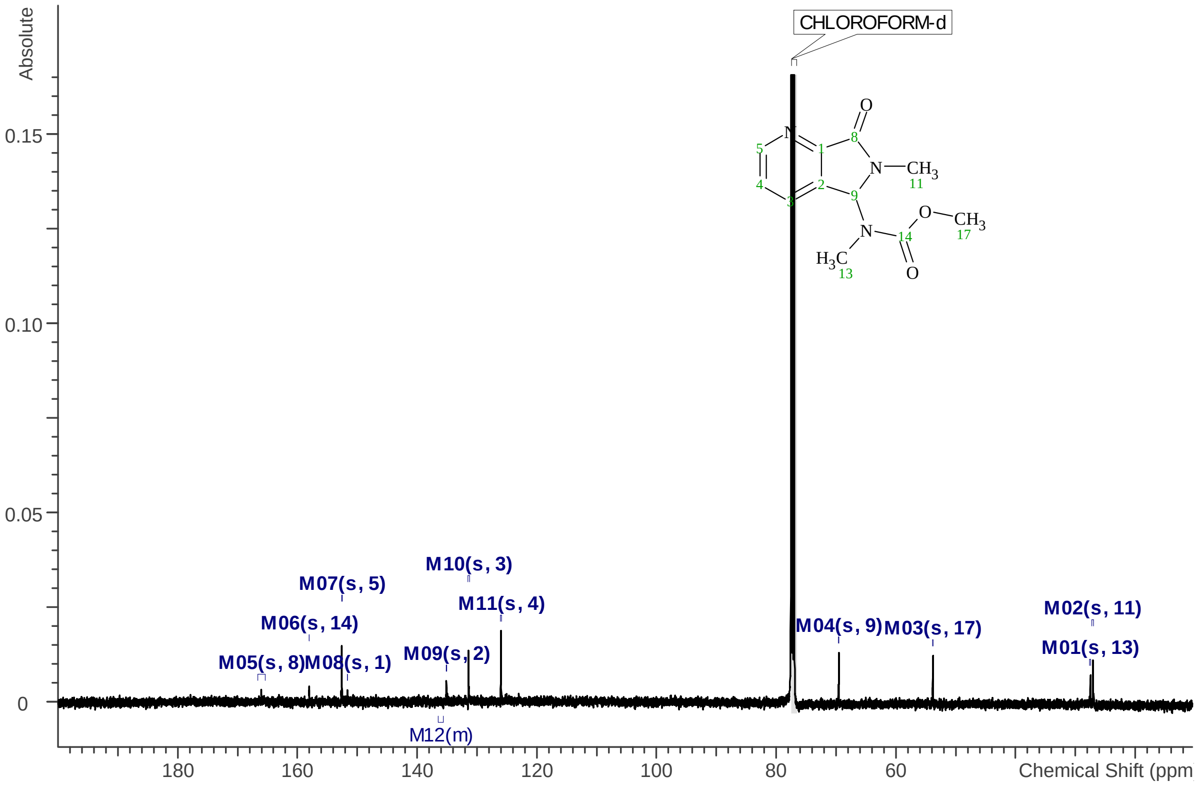
Absolute
CHLOROFORM-d
M05(s, 8)
M08(s, 1)
M06(s, 14)
M07(s, 5)
M09(s, 2)
M10(s, 3)
M11(s, 4)
M12(m)
M04(s, 9)
M03(s, 17)
M02(s, 11)
M01(s, 13)
Chemical Shift (ppm

8

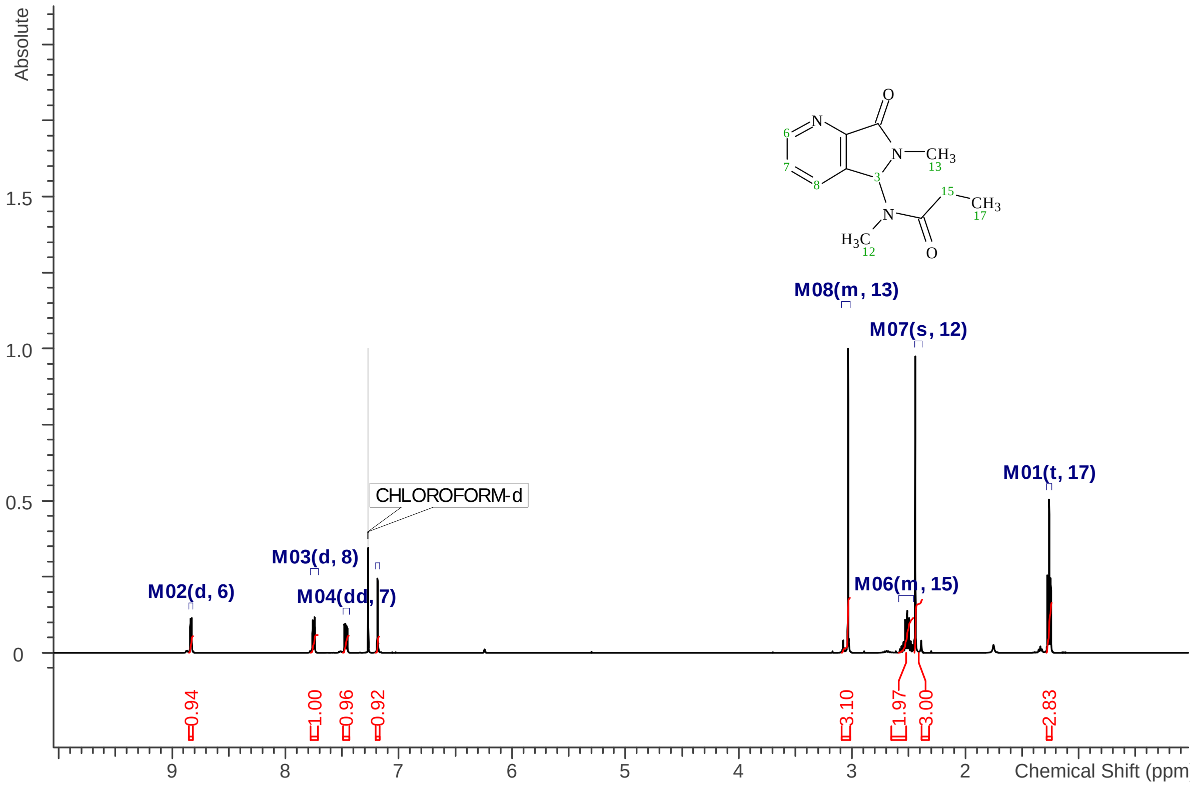
Absolute
M08(m, 13)
M07(s, 12)
M01(t, 17)
CHLOROFORM-d
M03(d, 8)
M02(d, 6)
M04(dd, 7)
M06(m, 15)
0.94
1.00
0.96
0.92
3.10
1.97
3.00
2.83
Chemical Shift (ppm

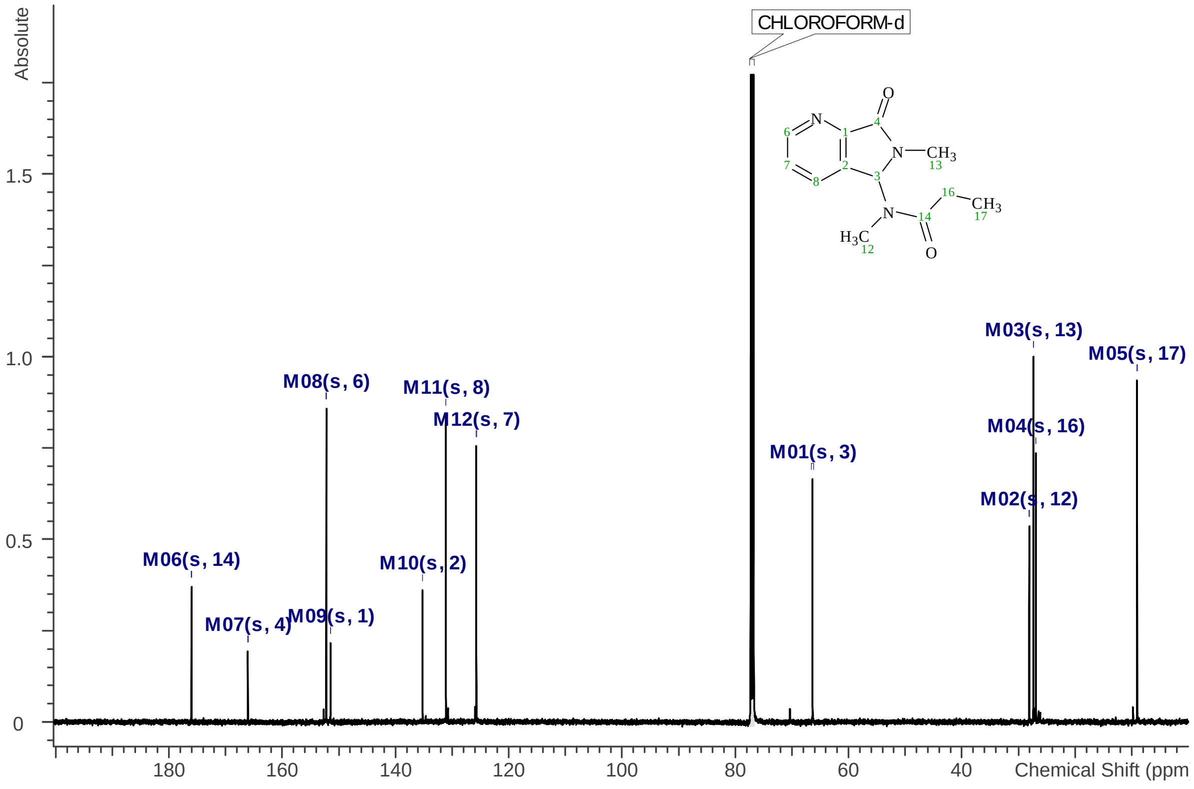
Absolute
CHLOROFORM-d
M03(s, 13)
M05(s, 17)
M08(s, 6)
M11(s, 8)
M12(s, 7)
M04(s, 16)
M01(s, 3)
M02(s, 12)
M06(s, 14)
M10(s, 2)
M07(s, 4)
M09(s, 1)
Chemical Shift (ppm

9

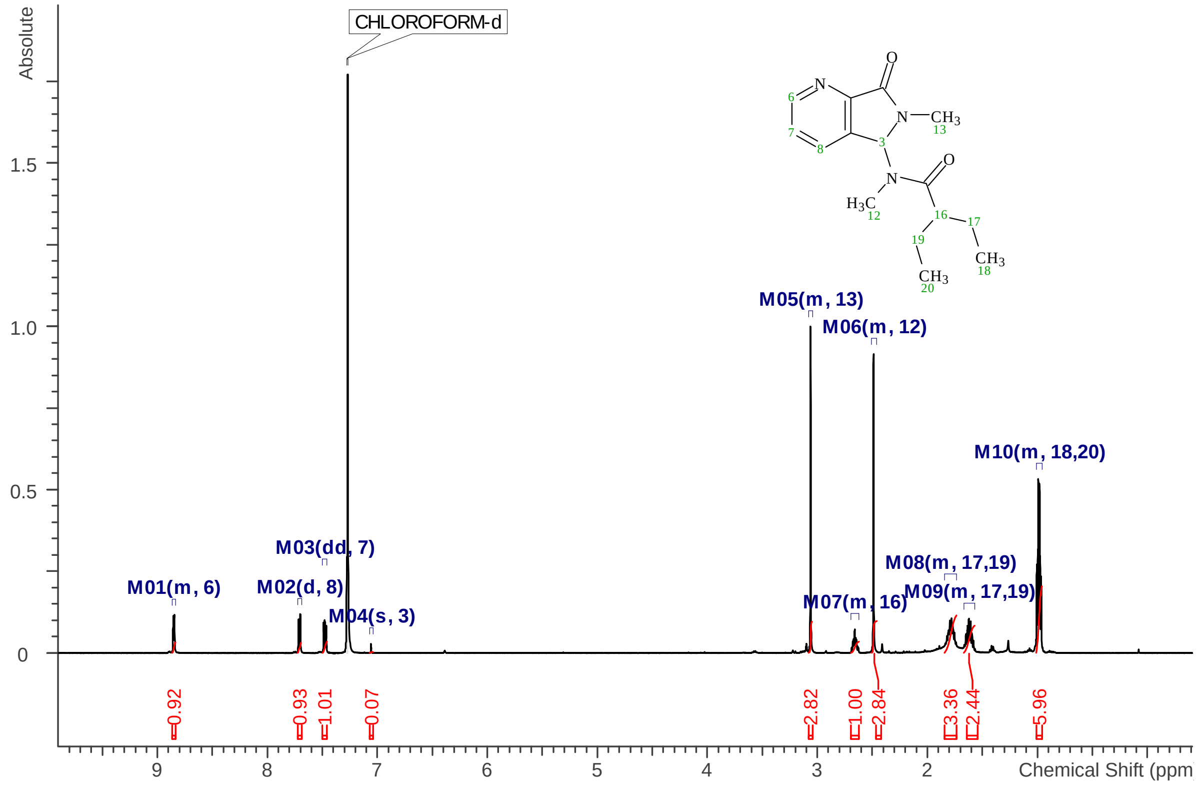
CHLOROFORM-d
M01(m, 6)
M02(d, 8)
M03(dd, 7)
M04(s, 3)
M05(m, 13)
M06(m, 12)
M07(m, 16)
M08(m, 17,19)
M09(m, 17,19)
M10(m, 18,20)
0.92
0.93
1.01
0.07
2.82
1.00
2.84
3.36
2.44
5.96
Absolute
Chemical Shift (ppm

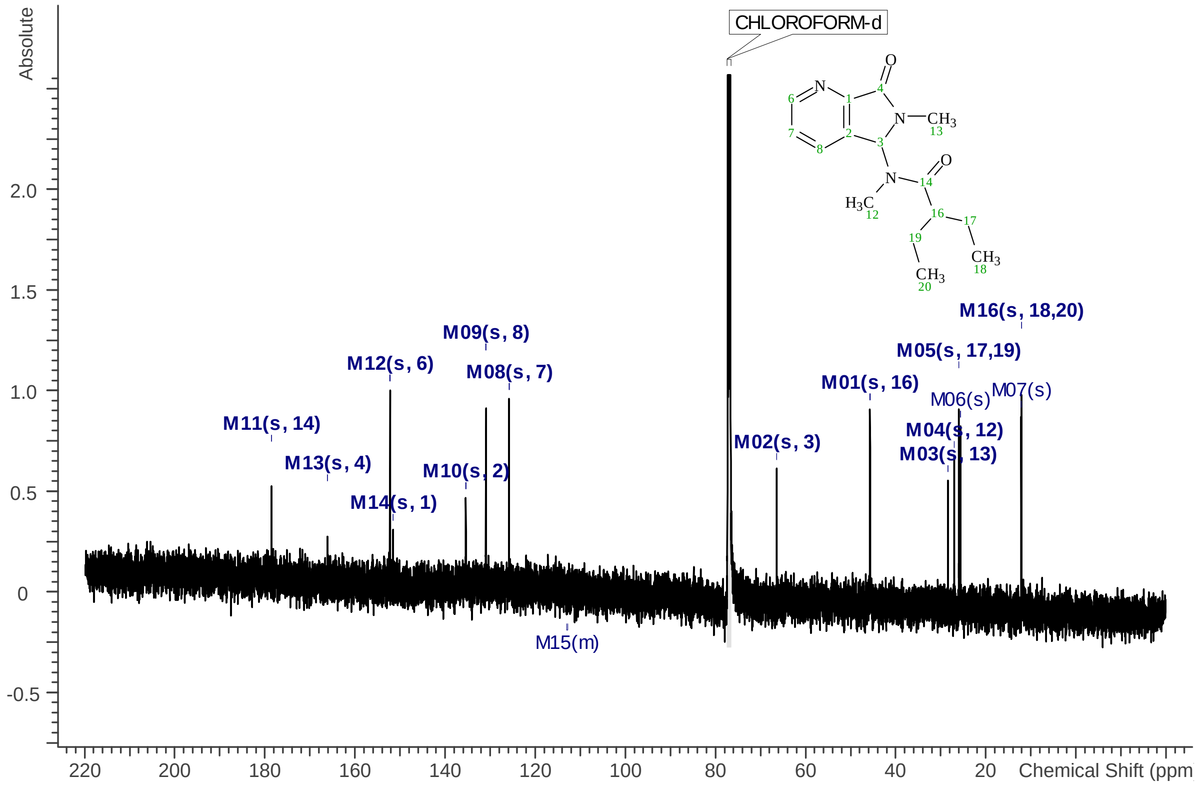
CHLOROFORM-d
M11(s, 14)
M13(s, 4)
M12(s, 6)
M14(s, 1)
M09(s, 8)
M10(s, 2)
M08(s, 7)
M15(m)
M02(s, 3)
M01(s, 16)
M05(s, 17,19)
M16(s, 18,20)
M06(s)
M07(s)
M04(s, 12)
M03(s, 13)
Absolute
Chemical Shift (ppm

10

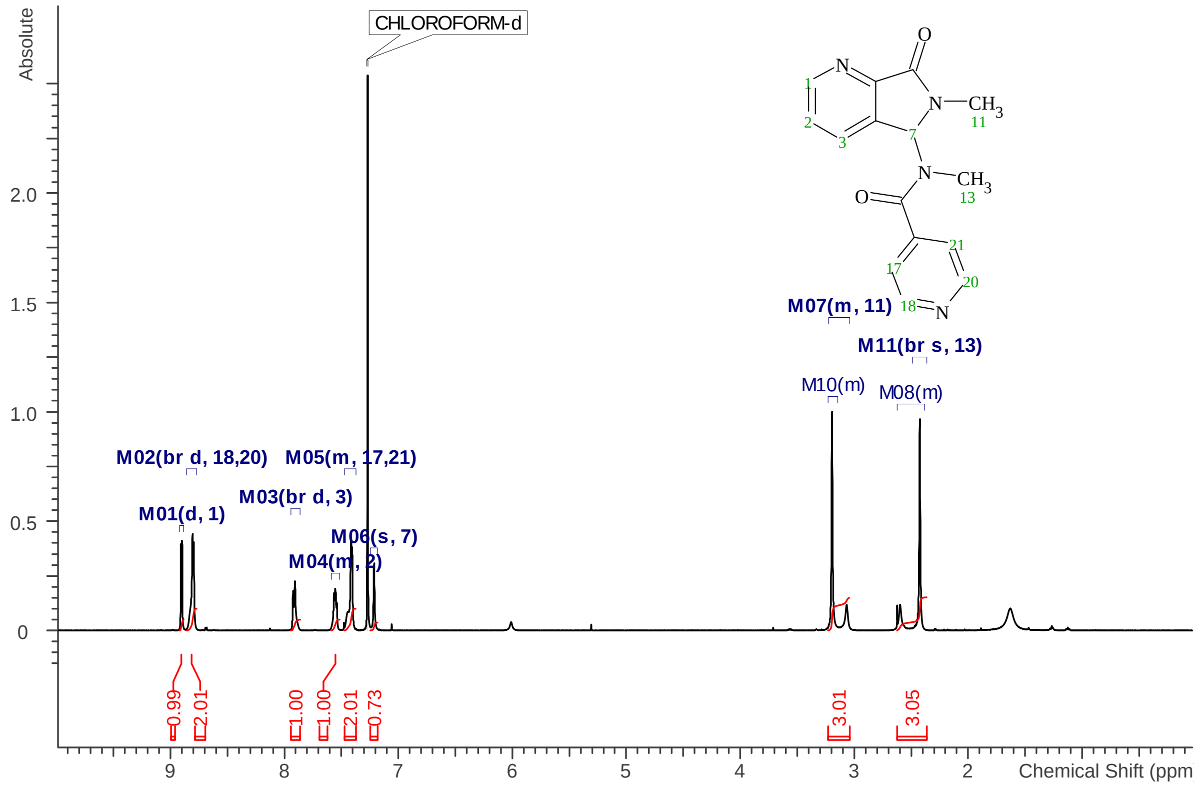
Absolute
CHLOROFORM-d
M01(d, 1)
M02(br d, 18,20)
M03(br d, 3)
M04(m, 2)
M05(m, 17,21)
M06(s, 7)
M07(m, 11)
M08(m)
M10(m)
M11(br s, 13)
0.99
2.01
1.00
1.00
2.01
0.73
3.01
3.05
Chemical Shift (ppm

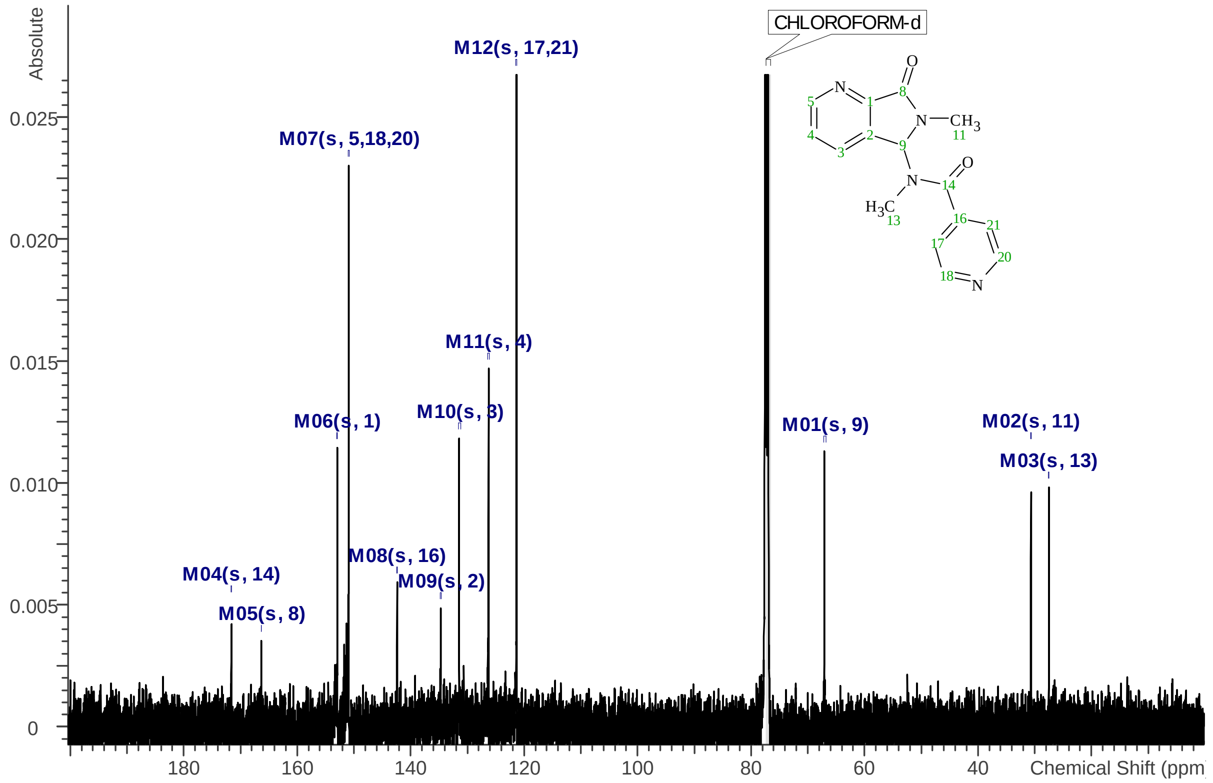
Absolute
CHLOROFORM-d
M01(s, 9)
M02(s, 11)
M03(s, 13)
M04(s, 14)
M05(s, 8)
M06(s, 1)
M07(s, 5,18,20)
M08(s, 16)
M09(s, 2)
M10(s, 3)
M11(s, 4)
M12(s, 17,21)
Chemical Shift (ppm

11 (please refer to page S36)

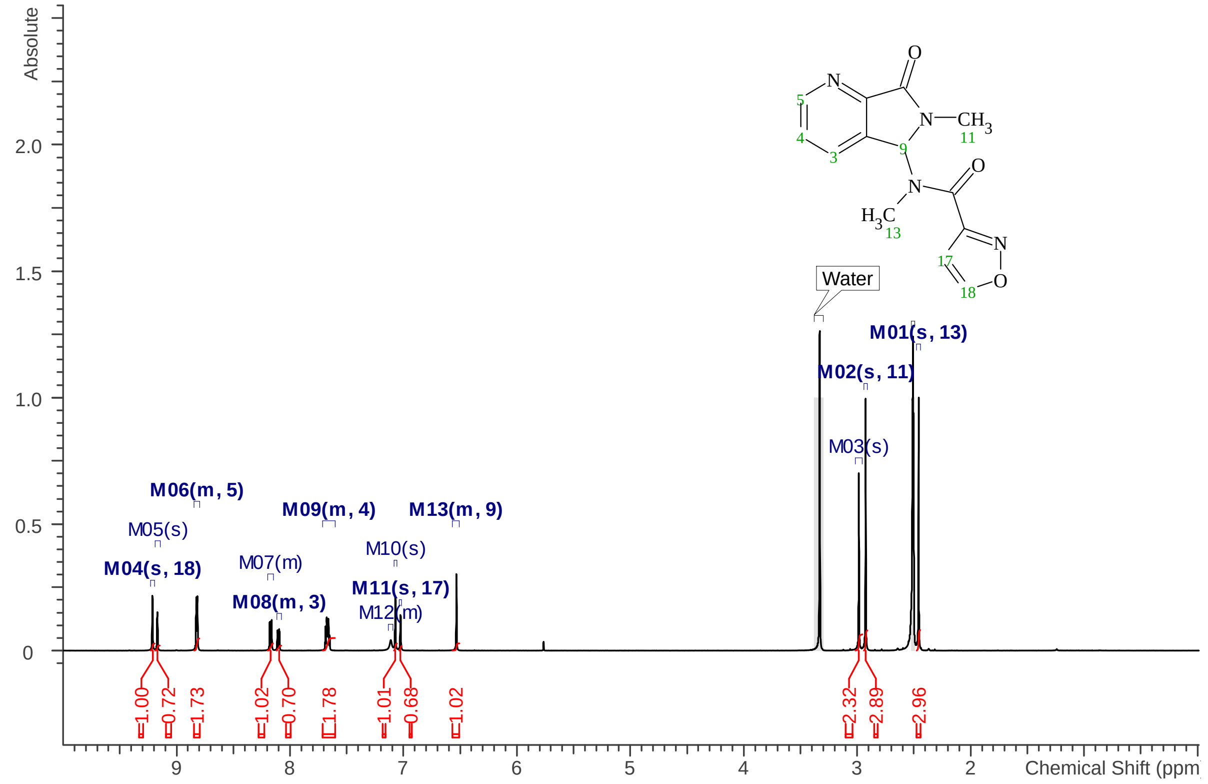

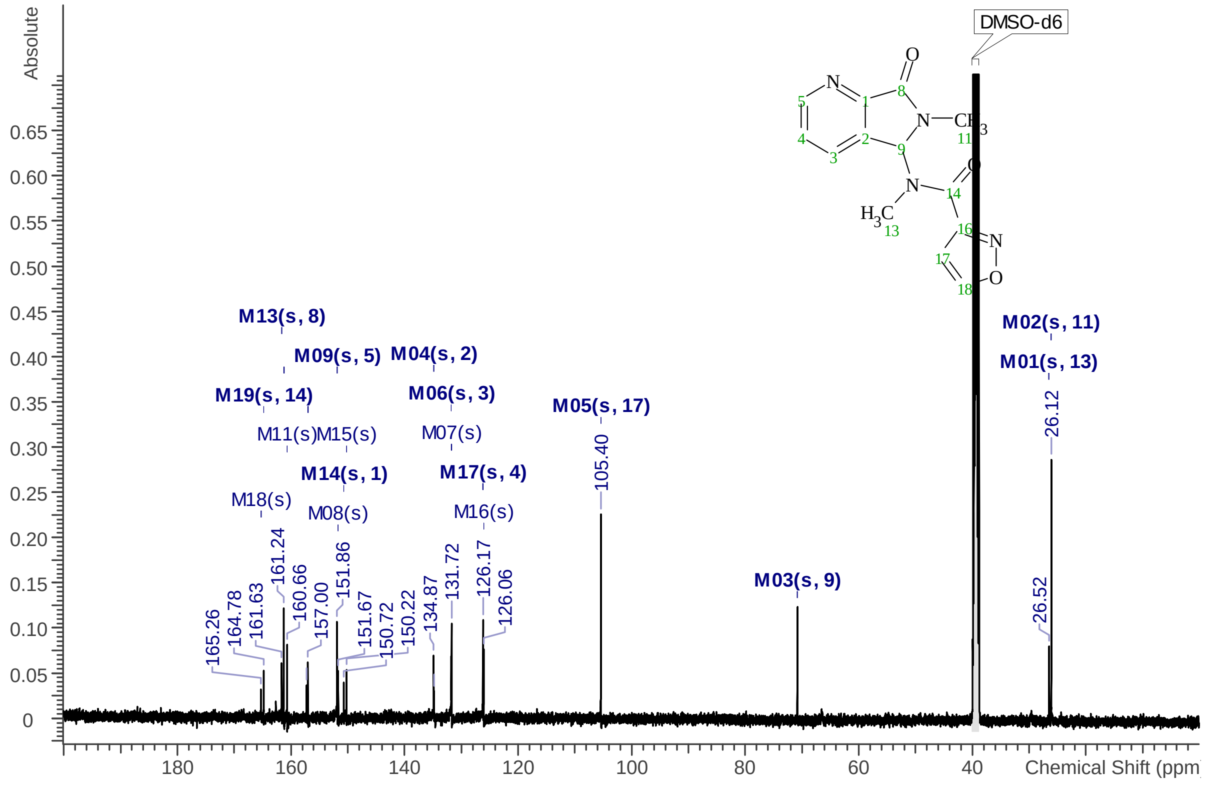

12

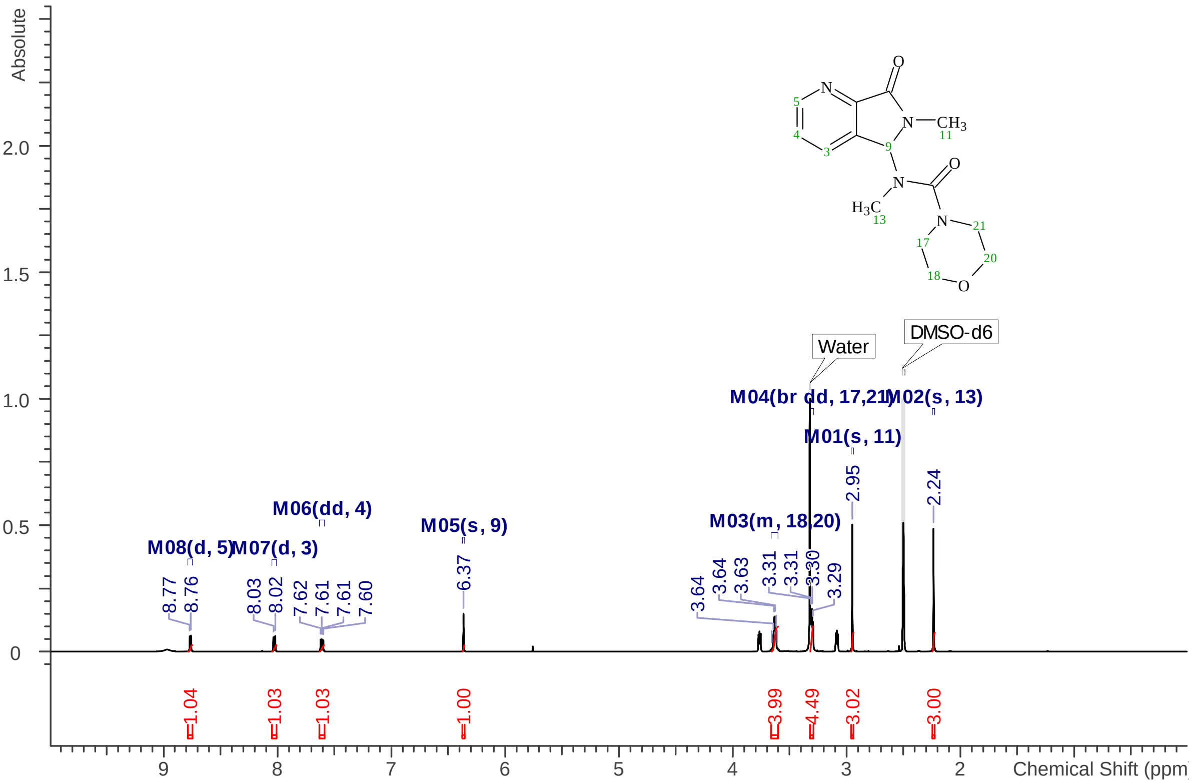
Absolute
Water
DMSO-d6
M08(d, 5)
M07(d, 3)
M06(dd, 4)
M05(s, 9)
M03(m, 18,20)
M04(br dd, 17,21)
M02(s, 13)
M01(s, 11)
8.77
8.76
8.03
8.02
7.62
7.61
7.61
7.60
6.37
3.64
3.64
3.63
3.31
3.31
3.30
3.29
2.95
2.24
1.04
1.03
1.03
1.00
3.99
4.49
3.02
3.00
Chemical Shift (ppm)

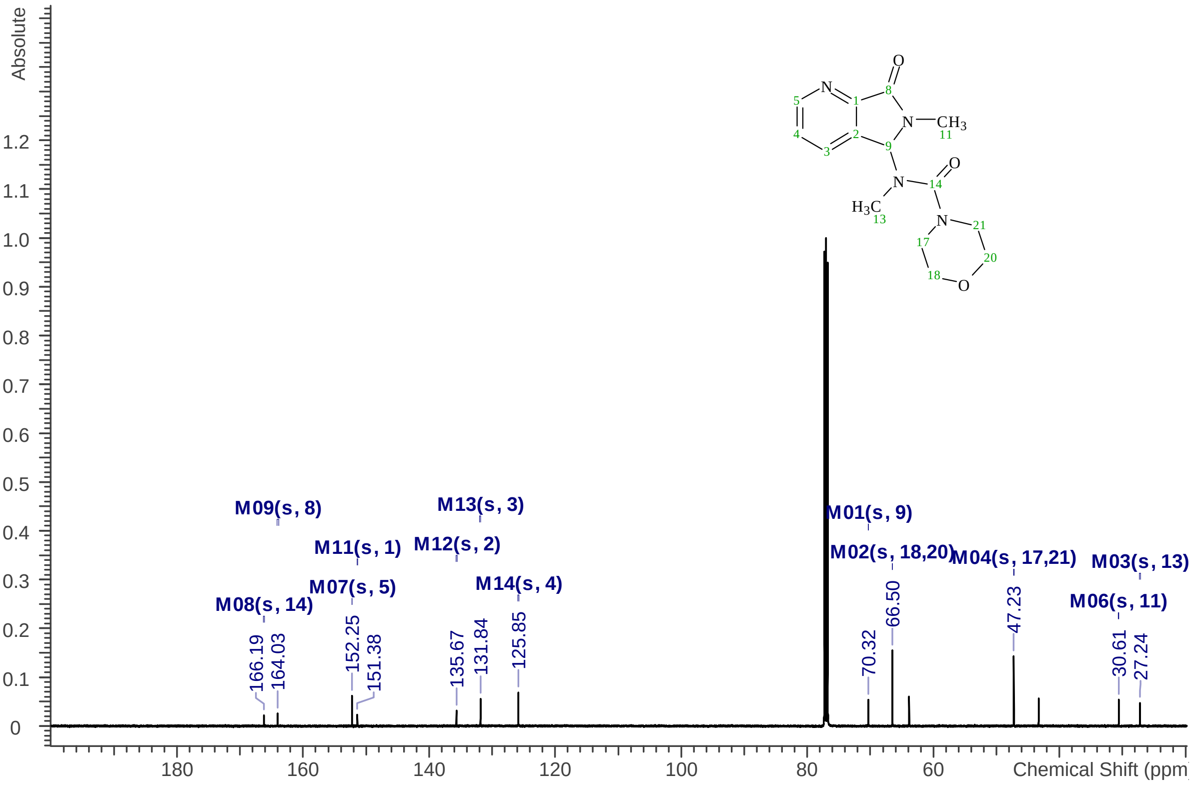
Absolute
M09(s, 8)
M13(s, 3)
M11(s, 1)
M12(s, 2)
M07(s, 5)
M14(s, 4)
M08(s, 14)
M01(s, 9)
M02(s, 18,20)
M04(s, 17,21)
M03(s, 13)
M06(s, 11)
166.19
164.03
152.25
151.38
135.67
131.84
125.85
70.32
66.50
47.23
30.61
27.24
Chemical Shift (ppm)

I

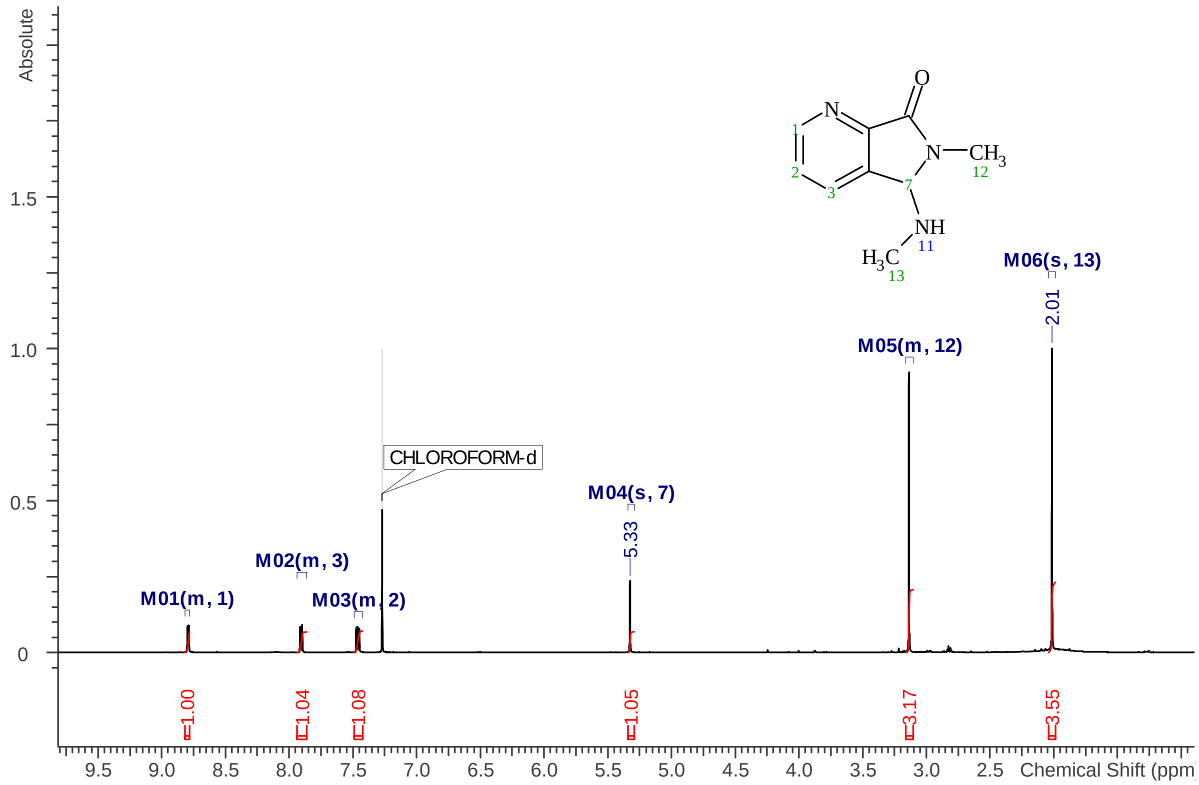

Absolute
CHLOROFORM-d
M01(m, 1)
M02(m, 3)
M03(m, 2)
M04(s, 7)
5.33
M05(m, 12)
M06(s, 13)
2.01
1.00
1.04
1.08
1.05
3.17
3.55
Chemical Shift (ppm

2

Mass Spectrum and Results

Injection Details

Injection Name:	**DWI-HIPS-8083**	*Run Time (min):*	**5,10**
Vial Number:	**GB8**	*Injection Volume:*	**2,00**
Injection Type:	**Unknown**	*Channel:*	**UV_VIS_1**
Calibration Level:		*Wavelength:*	**254**
Instrument Method:	**0.6ml_+ve_-ve_100-600**	*Bandwidth:*	**2**
Processing Method:	**Processing Method - New (190-380)**	*Dilution Factor:*	**1,0000**
Injection Date/Time:	**23.Jan.24 09:56**	*Sample Weight:*	**1,0000**

Integration Results

No.	Peak Name	Retention Time min	Area mAU*min	Height mAU	Relative Area %	Relative Height %	Amount n.a.
1		1,355	22,194	157,168	99,78	99,26	n.a.
2		2,244	0,050	1,173	0,22	0,74	n.a.
**Total:**			**22,244**	**158,341**	**100,00**	**100,00**	

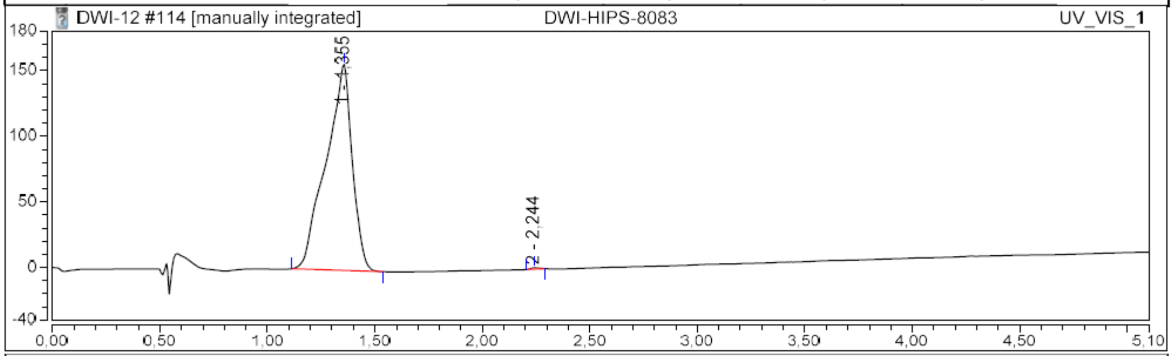

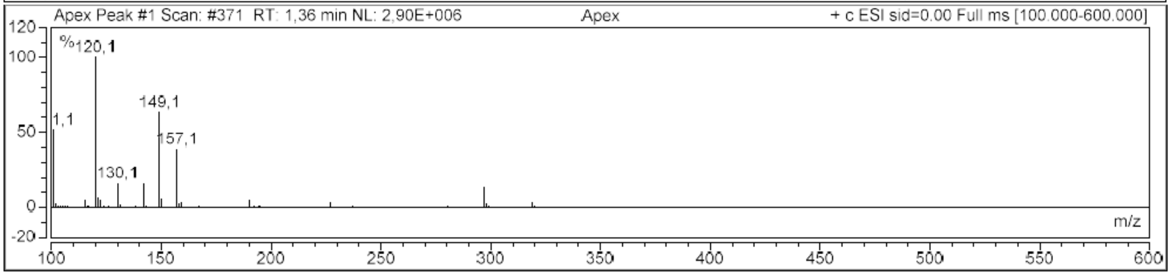

3

Mass Spectrum and Results

Injection Details

Injection Name:	**DWI-HIPS-7860**	*Run Time (min):*	**5.10**
Vial Number:	**GB6**	*Injection Volume:*	**2.00**
Injection Type:	**Unknown**	*Channel:*	**UV_VIS_1**
Calibration Level:		*Wavelength:*	**254**
Instrument Method:	**0.6ml_+ve_-ve_100-600**	*Bandwidth:*	**2**
Processing Method:	**Processing Method - New (190-380)**	*Dilution Factor:*	**1.0000**
Injection Date/Time:	**16/Jan/24 15:21**	*Sample Weight:*	**1.0000**

Integration Results

No.	Peak Name	Retention Time min	Area mAU*min	Height mAU	Relative Area %	Relative Height %	Amount n.a.
1		0.466	60.394	3609.390	96.83	98.28	n.a.
2		0.569	1.977	63.330	3.17	1.72	n.a.
**Total:**			**62.372**	**3672.720**	**100.00**	**100.00**	

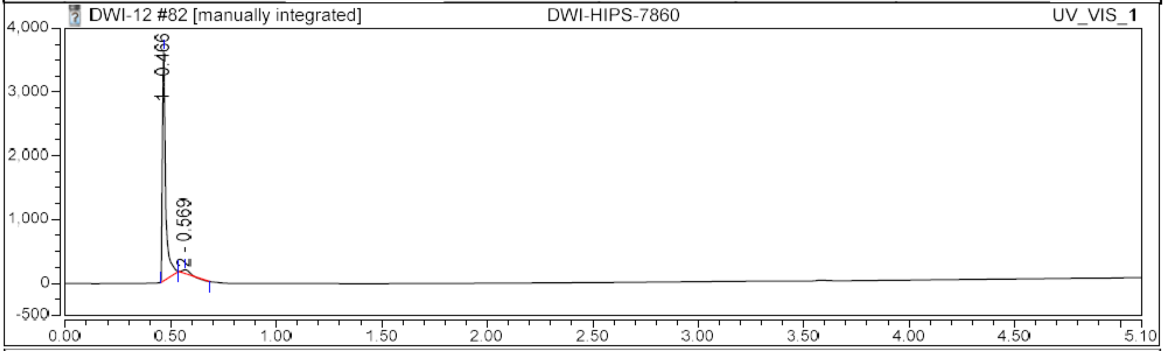

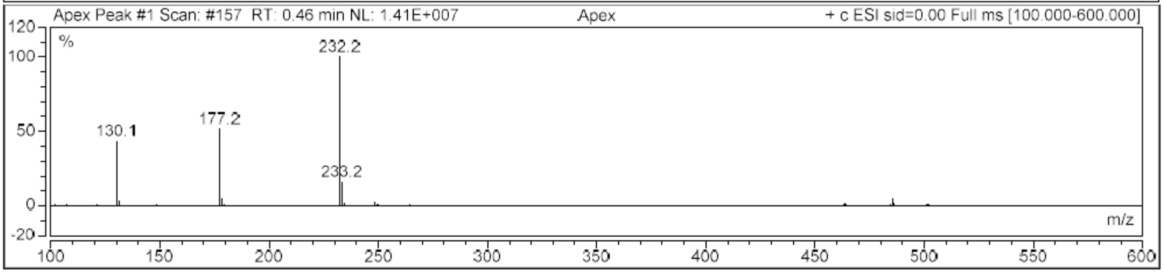

4

Mass Spectrum and Results

Injection Details

Injection Name:	**DWI-HIPS-7859**	*Run Time (min):*	**5.10**
Vial Number:	**GB6**	*Injection Volume:*	**2.00**
Injection Type:	**Unknown**	*Channel:*	**UV_VIS_1**
Calibration Level:		*Wavelength:*	**254**
Instrument Method:	**0.6ml_+ve_-ve_100-600**	*Bandwidth:*	**2**
Processing Method:	**Processing Method - 210-310nm**	*Dilution Factor:*	**1.0000**
Injection Date/Time:	**07/Mar/23 09:59**	*Sample Weight:*	**1.0000**

Integration Results

No.	Peak Name	Retention Time min	Area mAU*min	Height mAU	Relative Area %	Relative Height %	Amount n.a.
1		2.509	82.274	787.577	100.00	100.00	n.a.
**Total:**			**82.274**	**787.577**	**100.00**	**100.00**	

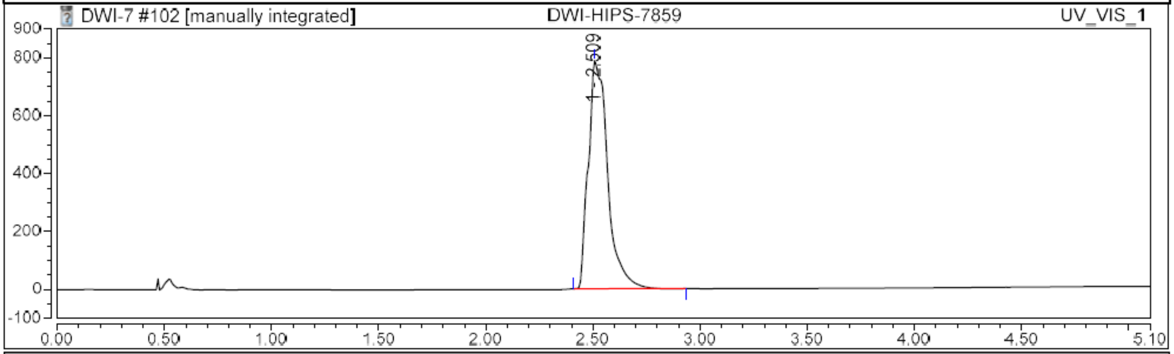

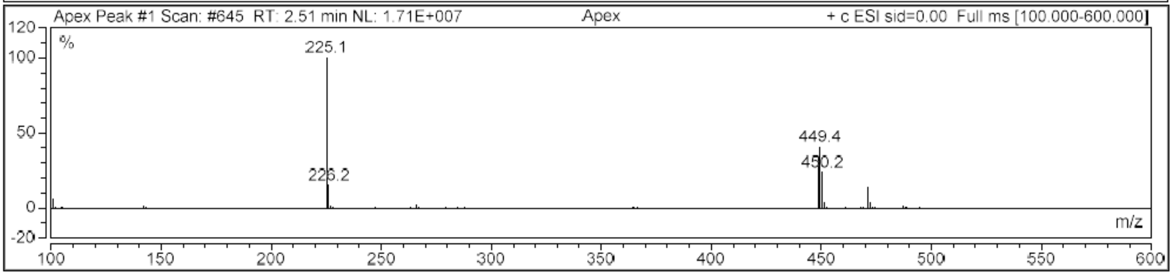

5

Mass Spectrum and Results

Injection Details

Injection Name:	**DWI-HIPS-7861**	*Run Time (min):*	**5.10**
Vial Number:	**GB7**	*Injection Volume:*	**2.00**
Injection Type:	**Unknown**	*Channel:*	**UV_VIS_1**
Calibration Level:		*Wavelength:*	**254**
Instrument Method:	**0.6ml_+ve_-ve_100-600**	*Bandwidth:*	**2**
Processing Method:	**Processing Method - New (190-380)**	*Dilution Factor:*	**1.0000**
Injection Date/Time:	**16/Jan/24 15:28**	*Sample Weight:*	**1.0000**

Integration Results

No.	Peak Name	Retention Time min	Area mAU*min	Height mAU	Relative Area %	Relative Height %	Amount n.a.
1		0.413	1.530	26.433	1.36	0.65	n.a.
2		0.462	111.278	4062.504	98.64	99.35	n.a.
**Total:**			**112.807**	**4088.937**	**100.00**	**100.00**	

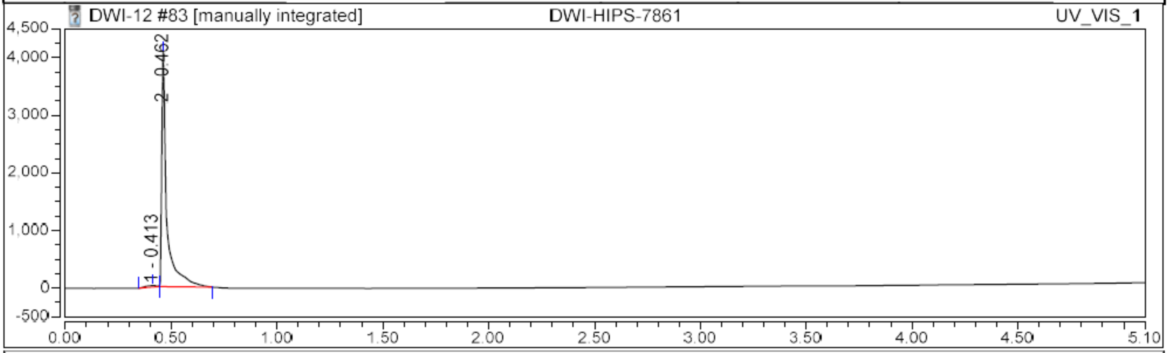

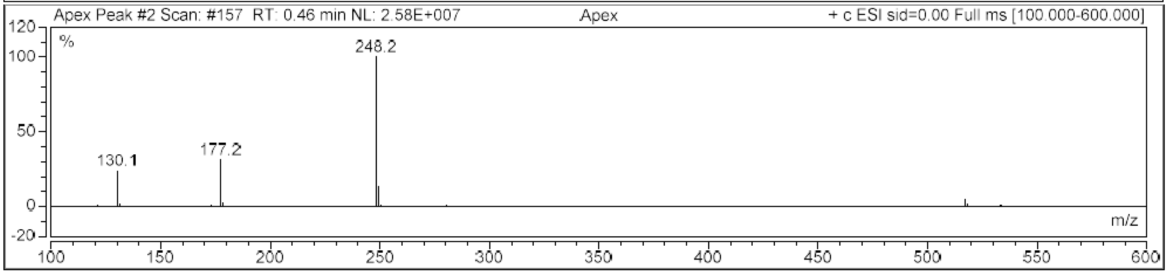

6

Mass Spectrum and Results

Injection Details			
Injection Name:	**DWI-HIPS-7858**	*Run Time (min):*	**5.10**
Vial Number:	**GB5**	*Injection Volume:*	**2.00**
Injection Type:	**Unknown**	*Channel:*	**UV_VIS_1**
Calibration Level:		*Wavelength:*	**254**
Instrument Method:	**0.6ml_+ve_-ve_100-600**	*Bandwidth:*	**2**
Processing Method:	**Processing Method - New (190-380)**	*Dilution Factor:*	**1.0000**
Injection Date/Time:	**16/Jan/24 15:14**	*Sample Weight:*	**1.0000**

Integration Results

No.	Peak Name	Retention Time min	Area mAU*min	Height mAU	Relative Area %	Relative Height %	Amount n.a.
1		2.435	36.513	675.647	97.25	98.17	n.a.
2		2.617	1.032	12.625	2.75	1.83	n.a.
**Total:**			**37.546**	**688.272**	**100.00**	**100.00**	

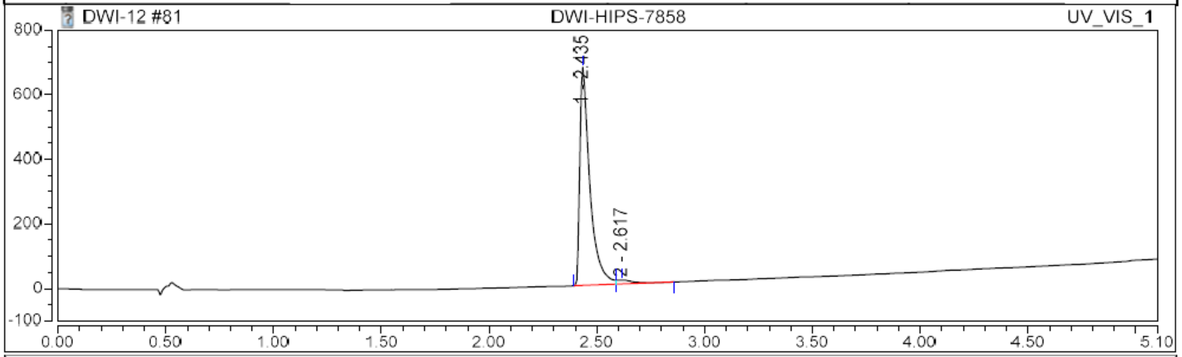

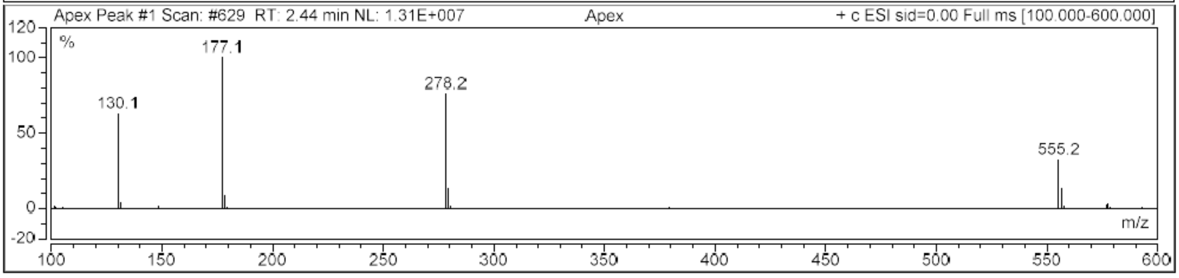

7

Mass Spectrum and Results

Injection Details

Injection Name:	**DWI-HIPS-8134**	*Run Time (min):*	**5,10**
Vial Number:	**GB6**	*Injection Volume:*	**2,00**
Injection Type:	**Unknown**	*Channel:*	**UV_VIS_1**
Calibration Level:		*Wavelength:*	**254**
Instrument Method:	**0.6ml_+ve_-ve_100-600**	*Bandwidth:*	**2**
Processing Method:	**Processing Method - New (190-380)**	*Dilution Factor:*	**1,0000**
Injection Date/Time:	**23.Jan.24 08:59**	*Sample Weight:*	**1,0000**

Integration Results

No.	Peak Name	Retention Time min	Area mAU*min	Height mAU	Relative Area %	Relative Height %	Amount n.a.
1		1,905	1,043	17,348	96,37	97,92	n.a.
2		3,648	0,039	0,369	3,63	2,08	n.a.
**Total:**			**1,082**	**17,716**	**100,00**	**100,00**	

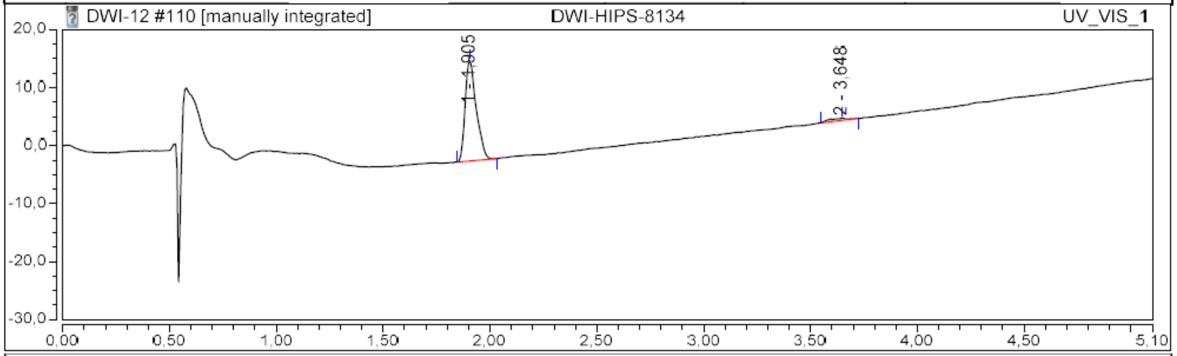

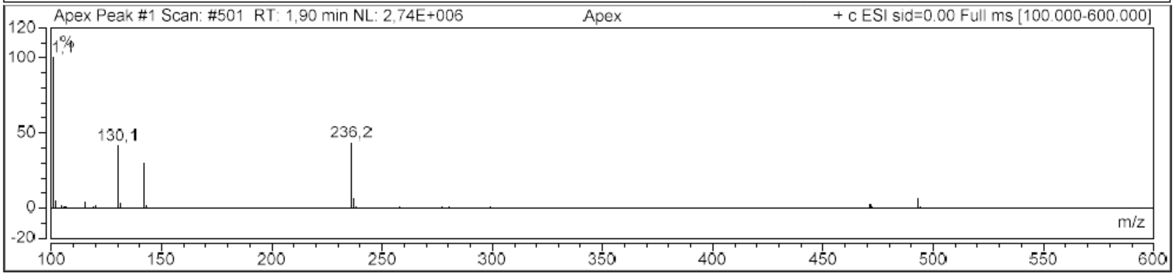

8

Mass Spectrum and Results

Injection Details

Injection Name:	**DWI-HIPS-8208**	*Run Time (min):*	**5.10**
Vial Number:	**GB5**	*Injection Volume:*	**2.00**
Injection Type:	**Unknown**	*Channel:*	**UV_VIS_1**
Calibration Level:		*Wavelength:*	**254**
Instrument Method:	**0.6ml_+ve_-ve_100-600**	*Bandwidth:*	**2**
Processing Method:	**Processing Method - New (190-380)**	*Dilution Factor:*	**1.0000**
Injection Date/Time:	**22/Jan/24 11:57**	*Sample Weight:*	**1.0000**

Integration Results

No.	Peak Name	Retention Time min	Area mAU*min	Height mAU	Relative Area %	Relative Height %	Amount n.a.
1		1.805	13.394	252.073	100.00	100.00	n.a.
**Total:**			**13.394**	**252.073**	**100.00**	**100.00**	

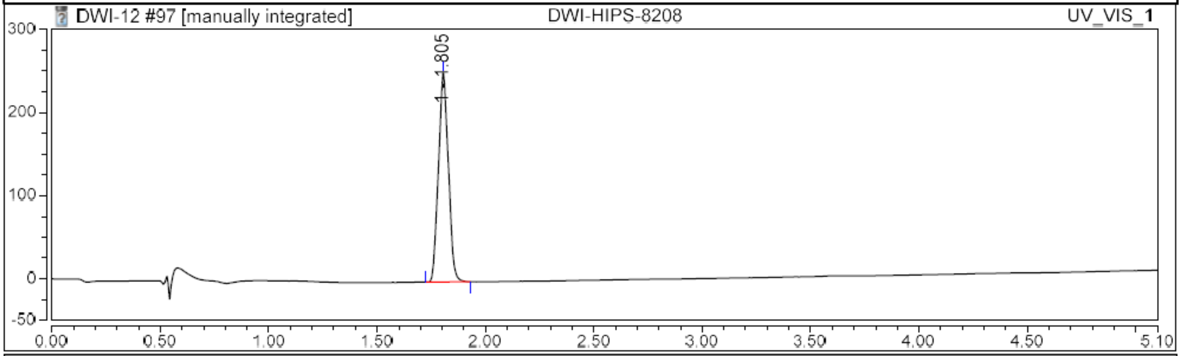

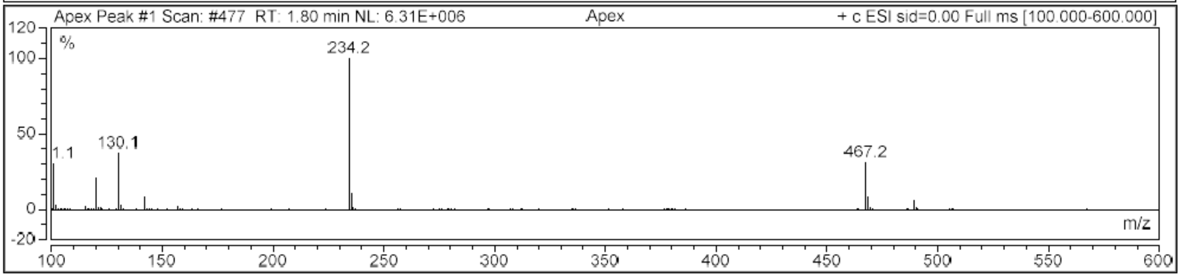

9

Mass Spectrum and Results

Injection Details

Injection Name:	**DWI-HIPS-8209**	*Run Time (min):*	**5.10**
Vial Number:	**GB6**	*Injection Volume:*	**2.00**
Injection Type:	**Unknown**	*Channel:*	**UV_VIS_1**
Calibration Level:		*Wavelength:*	**254**
Instrument Method:	**0.6ml_+ve_-ve_100-600**	*Bandwidth:*	**2**
Processing Method:	**Processing Method - New (190-380)**	*Dilution Factor:*	**1.0000**
Injection Date/Time:	**22/Jan/24 12:04**	*Sample Weight:*	**1.0000**

Integration Results

No.	Peak Name	Retention Time min	Area mAU*min	Height mAU	Relative Area %	Relative Height %	Amount n.a.
1		2.590	8.239	174.745	100.00	100.00	n.a.
**Total:**			**8.239**	**174.745**	**100.00**	**100.00**	

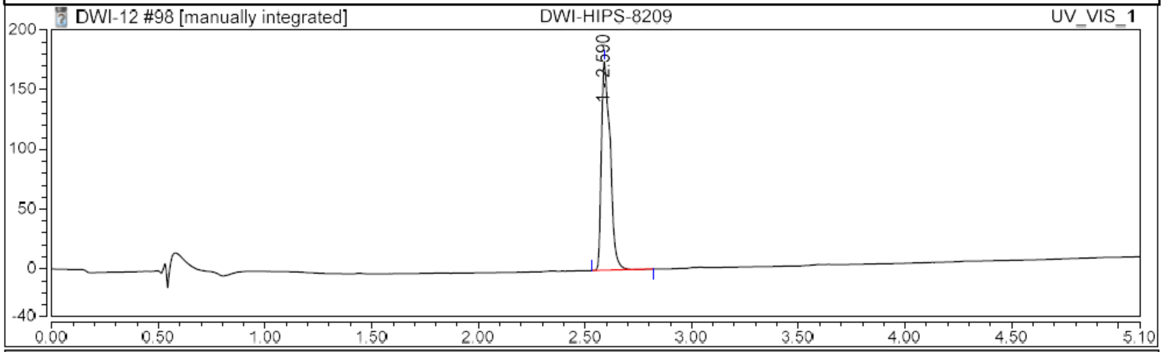

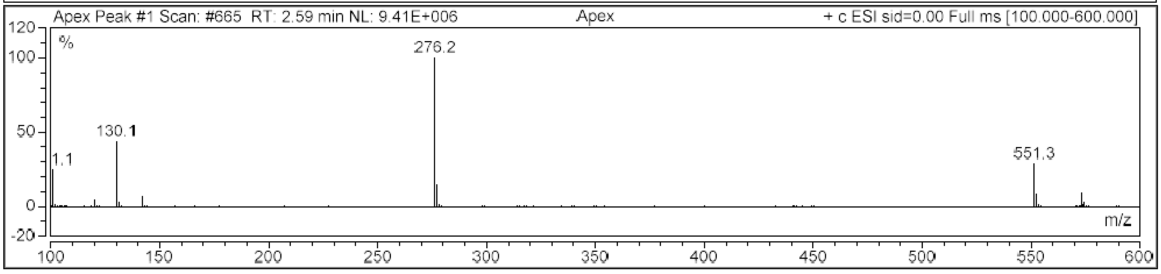

10

Mass Spectrum and Results

Injection Details

Injection Name:	**DWI-HIPS-8211**	*Run Time (min):*	**5.10**
Vial Number:	**GB7**	*Injection Volume:*	**2.00**
Injection Type:	**Unknown**	*Channel:*	**UV_VIS_1**
Calibration Level:		*Wavelength:*	**254**
Instrument Method:	**0.6ml_+ve_-ve_100-600**	*Bandwidth:*	**2**
Processing Method:	**Processing Method - New (190-380)**	*Dilution Factor:*	**1.0000**
Injection Date/Time:	**22/Jan/24 12:11**	*Sample Weight:*	**1.0000**

Integration Results

No.	Peak Name	Retention Time min	Area mAU*min	Height mAU	Relative Area %	Relative Height %	Amount n.a.
1		1.528	25.435	291.262	100.00	100.00	n.a.
**Total:**			**25.435**	**291.262**	**100.00**	**100.00**	

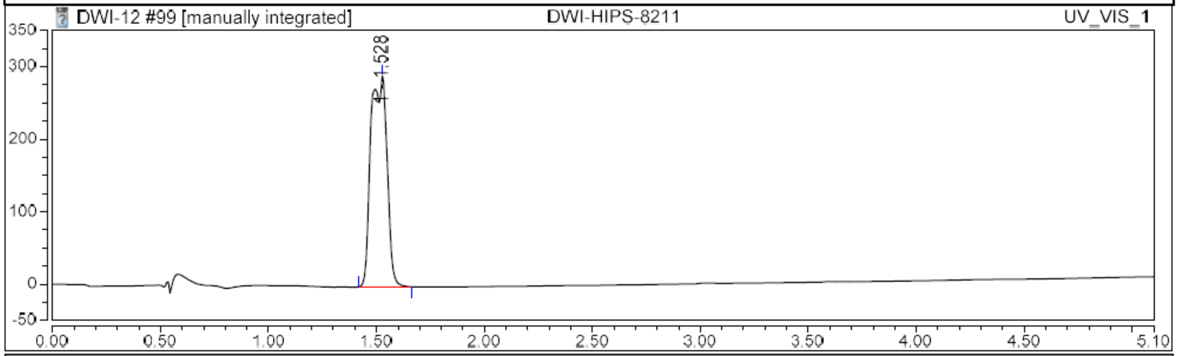

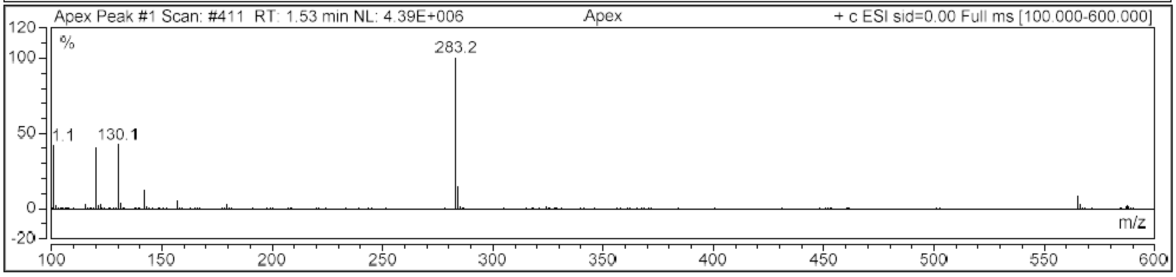

11

Mass Spectrum and Results

Injection Details

Injection Name:	**DWI-HIPS-8213**	*Run Time (min):*	**5.10**
Vial Number:	**GB5**	*Injection Volume:*	**2.00**
Injection Type:	**Unknown**	*Channel:*	**UV_VIS_1**
Calibration Level:		*Wavelength:*	**254**
Instrument Method:	**0.6ml_+ve_-ve_100-600**	*Bandwidth:*	**2**
Processing Method:	**Processing Method - New (190-380)**	*Dilution Factor:*	**1.0000**
Injection Date/Time:	**22/Jan/24 13:05**	*Sample Weight:*	**1.0000**

Integration Results

No.	Peak Name	Retention Time min	Area mAU*min	Height mAU	Relative Area %	Relative Height %	Amount n.a.
1		2.408	12.451	266.984	100.00	100.00	n.a.
**Total:**			**12.451**	**266.984**	**100.00**	**100.00**	

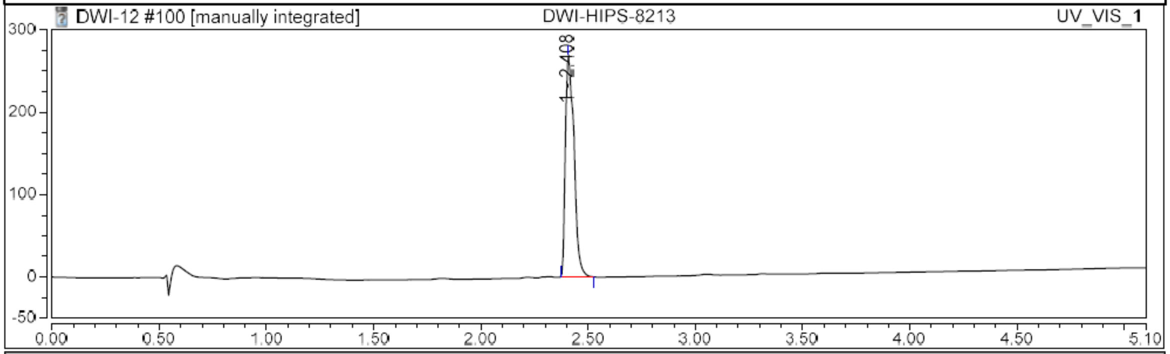

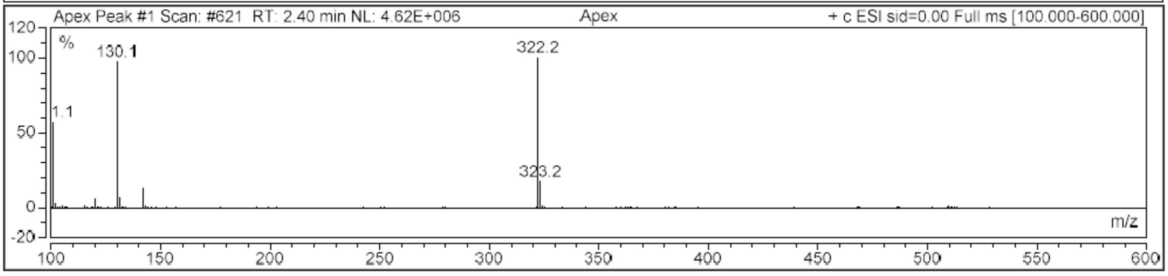

12

Mass Spectrum and Results

Injection Details

Injection Name:	**DWI-HIPS-8246**	*Run Time (min):*	**5.10**
Vial Number:	**GB7**	*Injection Volume:*	**2.00**
Injection Type:	**Unknown**	*Channel:*	**UV_VIS_1**
Calibration Level:		*Wavelength:*	**254**
Instrument Method:	**0.6ml_+ve_-ve_100-600**	*Bandwidth:*	**2**
Processing Method:	**Processing Method - New (190-380)**	*Dilution Factor:*	**1.0000**
Injection Date/Time:	**22/Jan/24 13:19**	*Sample Weight:*	**1.0000**

Integration Results

No.	Peak Name	Retention Time min	Area mAU*min	Height mAU	Relative Area %	Relative Height %	Amount n.a.
1		1.830	5.528	108.041	98.01	98.06	n.a.
2		3.043	0.090	1.543	1.59	1.40	n.a.
3		3.310	0.023	0.599	0.41	0.54	n.a.
**Total:**			**5.641**	**110.182**	**100.00**	**100.00**	

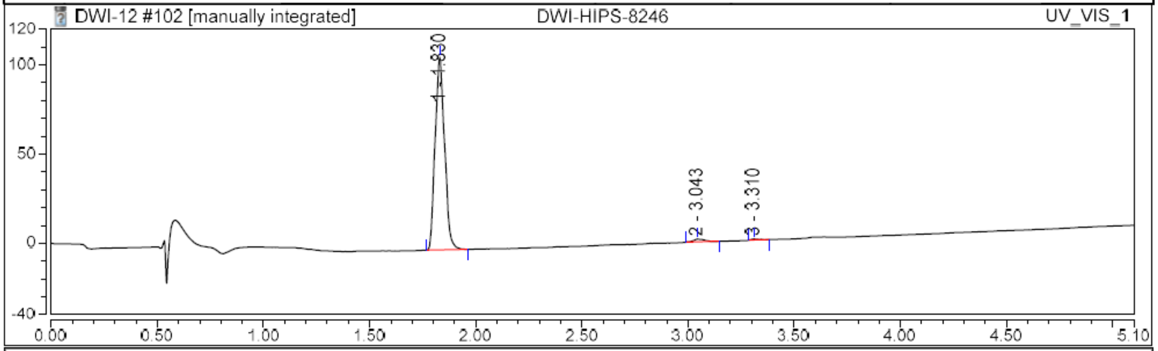

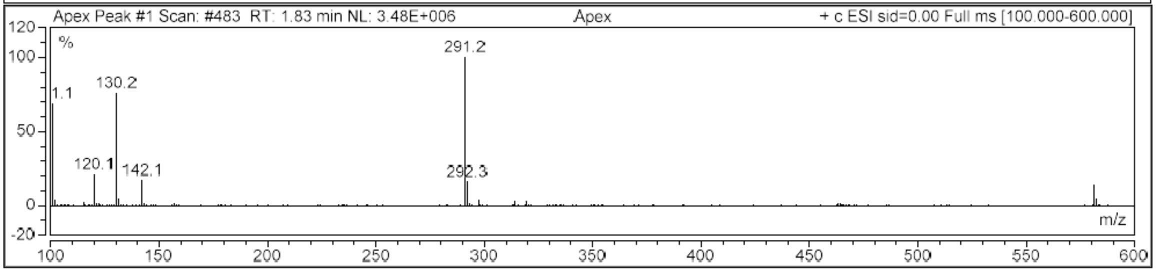

I

Mass Spectrum and Results

Injection Details

Injection Name:	**DWI-HIPS-8133**	*Run Time (min):*	**5.10**
Vial Number:	**GB8**	*Injection Volume:*	**2.00**
Injection Type:	**Unknown**	*Channel:*	**UV_VIS_1**
Calibration Level:		*Wavelength:*	**254**
Instrument Method:	**0.6ml_+ve_-ve_100-600**	*Bandwidth:*	**2**
Processing Method:	**Processing Method - New (190-380)**	*Dilution Factor:*	**1.0000**
Injection Date/Time:	**10/Jan/24 11:40**	*Sample Weight:*	**1.0000**

Integration Results

No.	Peak Name	Retention Time min	Area mAU*min	Height mAU	Relative Area %	Relative Height %	Amount n.a.
1		0.483	21.783	1492.236	75.82	87.82	n.a.
2		0.635	6.946	207.045	24.18	12.18	n.a.
**Total:**			**28.729**	**1699.281**	**100.00**	**100.00**	

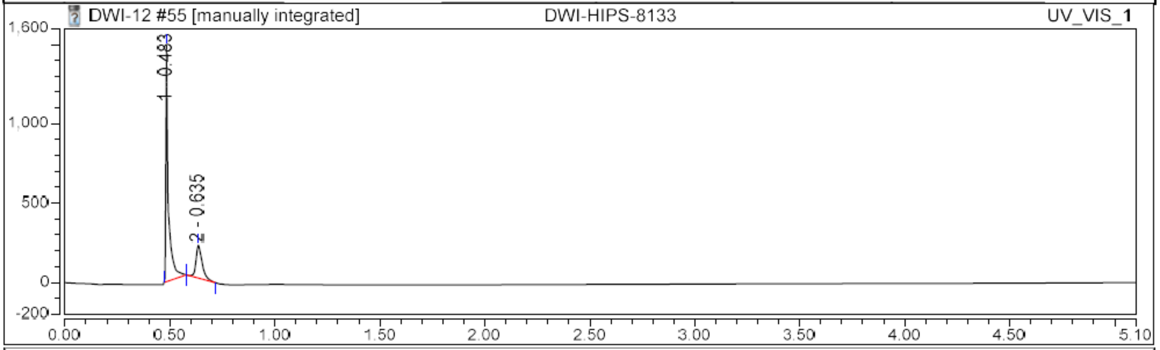

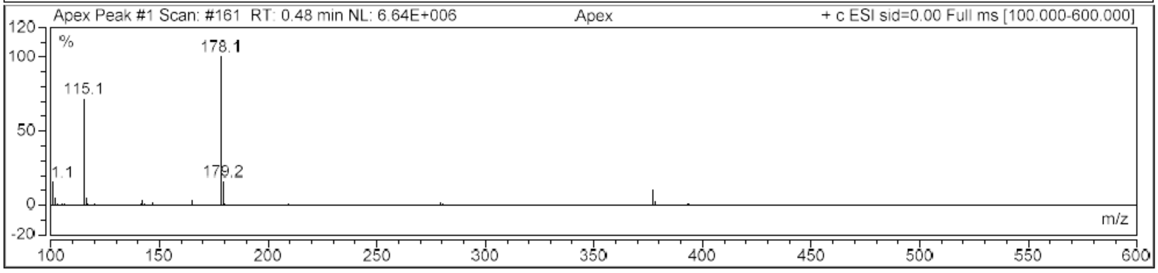

Heated NMR Spectra of 12

To validate our suspicion that the extra peaks in the NMR spectrum of **12** are due to rotamerisation, we measured both ^{1}H and ^{13}C at elevated temperatures (60 and 80 °C). As can be seen below, the “double peaks” converge at elevated temperatures confirming our suspicion.

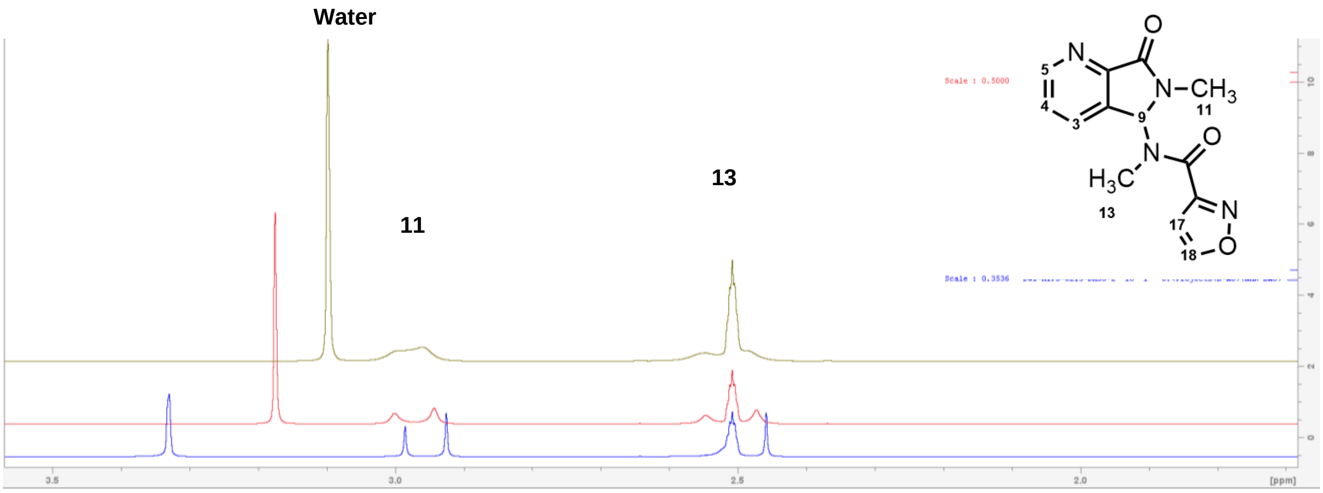

Figure S4 Comparison of ^{1}H NMR spectra of **12** at various temperatures from 1.5 to 3.5 ppm. Bottom: Spectrum measured at 25 °C. Middle: Spectrum measured at 60 °C. ¨Top: Spectrum measured at 80 °C

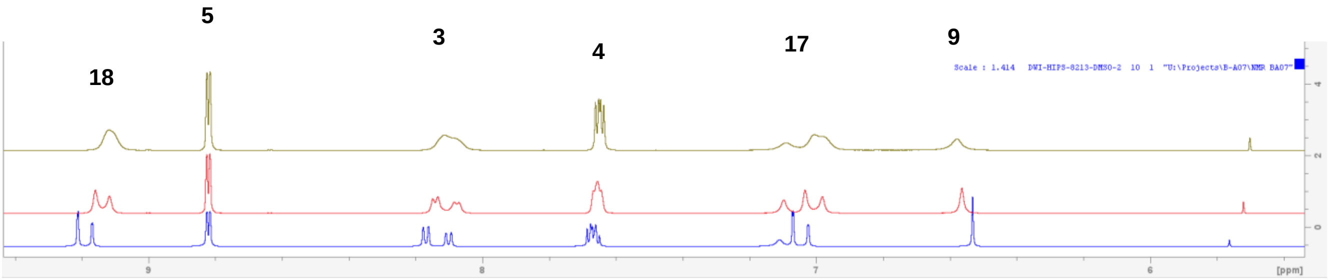

Figure S5 Comparison of ^{1}H NMR spectra of **12** at various temperatures from 5.5 to 9.5 ppm. Bottom: Spectrum measured at 25 °C. Middle: Spectrum measured at 60 °C. ¨Top: Spectrum measured at 80 °C

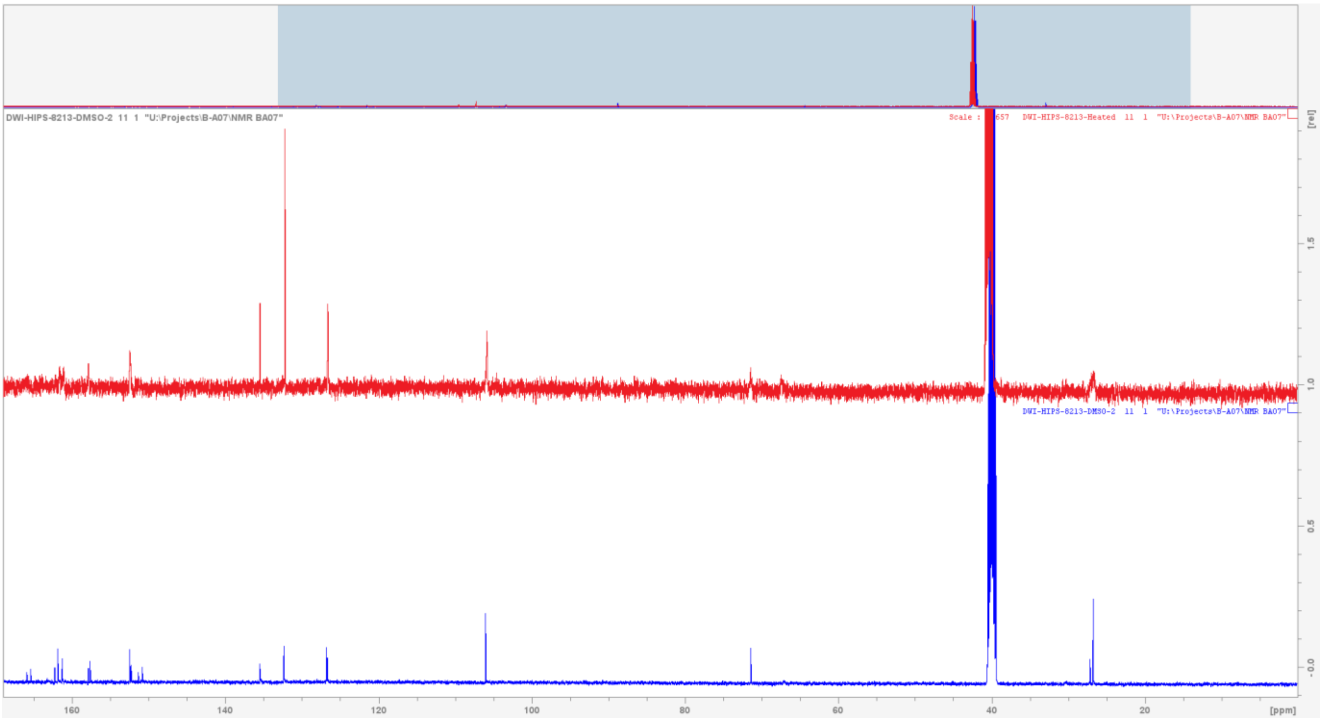

Figure S6 Comparison of ^{13}C NMR spectra of **12** at various temperatures. Bottom: Spectrum measured at 25 °C. Top: Spectrum measured at 80 °C

References

[15] S. B. Richard, M. E. Bowman, W. Kwiatkowski, I. Kang, C. Chow, A. M. Lillo, D. E. Cane, J. P. Noel, *Nature Structural Biology* **2001**, *8*, 641-648.

[16] P. V. Afonine, R. W. Grosse-Kunstleve, N. Echols, J. J. Headd, N. W. Moriarty, M. Mustyakimov, T. C. Terwilliger, A. Urzhumtsev, P. H. Zwart, P. D. Adams, *Acta Crystallographica Section D: Biological Crystallography* **2012**, *68*, 352-367; A. J. McCoy, R. W. Grosse-Kunstleve, P. D. Adams, M. D. Winn, L. C. Storoni, R. J. Read, *Journal of applied crystallography* **2007**, *40*, 658-674; W. Kabsch, A. C. Xds, *Crystallogr* **2010**, *66*, 125-132; P. Emsley, B. Lohkamp, W. G. Scott, K. Cowtan, *Acta Crystallographica Section D: Biological Crystallography* **2010**, *66*, 486-501.

[17] D. Willocx, L. Bizzarri, A. Alhayek, D. Kannan, P. Bravo, B. Illarionov, K. Rox, J. Lohse, M. Fischer, A. M. Kany, H. Hahne, M. Rottmann, M. Witschel, A. Odom John, M. M. Hamed, E. Diamanti, A. K. H. Hirsch, *Journal of Medicinal Chemistry* **2024**.
